# Collaboration between Antagonistic Cell Type Regulators Governs Natural Variation in the Candida albicans Biofilm and Hyphal Gene Expression Network

**DOI:** 10.1128/mbio.01937-22

**Published:** 2022-08-22

**Authors:** Eunsoo Do, Max V. Cravener, Manning Y. Huang, Gemma May, C. Joel McManus, Aaron P. Mitchell

**Affiliations:** a Department of Microbiology, University of Georgia, Athens, Georgia, USA; b Department of Biological Sciences, Carnegie Mellon University, Pittsburgh, Pennsylvania, USA; Yonsei University

**Keywords:** biofilm, *Candida albicans*, gene regulation, hyphae, transcription

## Abstract

Candida albicans is among the most significant human fungal pathogens. However, the vast majority of C. albicans studies have focused on a single clinical isolate and its marked derivatives. We investigated natural variation among clinical C. albicans isolates in gene regulatory control of biofilm formation, a process crucial to virulence. The transcription factor Efg1 is required for biofilm-associated gene expression and biofilm formation. Previously, we found extensive variation in Efg1-responsive gene expression among 5 diverse clinical isolates. However, chromatin immunoprecipitation sequencing analysis showed that Efg1 binding to genomic loci was uniform among the isolates. Functional dissection of strain differences identified three transcription factors, Brg1, Tec1, and Wor1, for which small changes in expression levels reshaped the Efg1 regulatory network. Brg1 and Tec1 are known biofilm activators, and their role in Efg1 network variation may be expected. However, Wor1 is a known repressor of *EFG1* expression and an inhibitor of biofilm formation. In contrast, we found that a modest increase in *WOR1* RNA levels, reflecting the expression differences between C. albicans strains, could augment biofilm formation and expression of biofilm-related genes. The analysis of natural variation here reveals a novel function for a well-characterized gene and illustrates that strain diversity offers a unique resource for elucidation of network interactions.

## INTRODUCTION

Candida albicans is among the most significant human fungal pathogens. It can grow in multiple cell morphologies, including yeast and hyphae. Yeasts are ovoid cells that divide by budding; hyphae are long tubular cell arrays that grow by tip extension. Hyphae are required for host cell damage and virulence in diverse animal infection models. Hyphae are also required to form stable biofilms, the cause of device-associated infections. Therefore, the yeast-hyphal switch is considered central to C. albicans infection biology.

Hyphal growth is accompanied by expression of hypha-associated genes, whose products function in adhesion, tissue damage, invasion, and other pathogenic processes (1, 2). Hypha-associated gene expression is governed by a network of transcription factors (TFs) that are interconnected through shared target genes and the control of one another’s expression (3). Efg1 is among the most well-studied TFs in this network. It is required for expression of hypha-associated genes and for formation of hyphae and biofilm under most conditions (4). Efg1 is also required for virulence in many animal infection models (4).

Efg1 has been called a master regulator (1, 5–7), because it controls a second cell morphogenic switch, the white-opaque switch, in which ovoid yeast cells (called “white” cells in this context) switch to an elongated “opaque” cell type that can engage in mating. The white-opaque switch is activated by a second master regulator, Wor1, and its associated transcription factor network (8). Efg1 and Wor1 are mutually antagonistic: high levels of Efg1 repress *WOR1* expression, and high levels of Wor1 repress *EFG1* expression (7, 8). The balance of Efg1 and Wor1 levels can enable either the yeast-to-hypha switch or the white-to-opaque switch (1, 5–7).

Antagonism between Efg1 and Wor1 is also the basis for their roles in gut commensalism (7, 9). Loss of *EFG1* activity, via mutation of *EFG1* itself or through overexpression of *WOR1*, favors elevated colonization levels in the murine gastrointestinal tract. Conversely, loss of *WOR1* leads to reduced colonization levels. Colonizing cells with sustained overexpression of *WOR1* undergo a morphogenic transition from white to GUT cells (gastrointestinally induced transition cells) and have a gene expression profile suggestive of metabolic adaptation to the gastrointestinal tract (7, 9). These studies have shown that Wor1 function extends beyond mating, and they reinforce the principle that Efg1 and Wor1 achieve their biological impacts through mutual inhibition.

Recent studies have shown that there is a second kind of interaction between Efg1 and Wor1 (10). The two proteins can form phase-separated condensates *in vitro* and when expressed in human cells. Condensate formation depends upon a Wor1 prion-like domain (PrLD) and is abolished by two different sets of amino acid substitutions that affect acidic PrLD residues (10). Wor1 PrLD mutant proteins are unable to promote white-opaque switching, thus arguing that condensate formation is required for a known function of Wor1.

The understanding of Efg1 and the biofilm and hyphal regulatory network reflects almost entirely the features of one strain of C. albicans, SC5314, and its auxotrophic derivatives. C. albicans clinical isolates differ in properties that are under Efg1 control, including capacity to form hyphae and biofilm (11, 12). Indeed, Efg1-responsive gene expression varies considerably among C. albicans clinical isolates; RNA sequencing (RNA-seq) analysis in five clinical isolates showed that ~700 genes presented significant expression differences in response to *efg1*Δ/Δ mutations in each strain background, yet only a fraction of those genes presented expression differences in all five clinical isolates (13). How is it that the Efg1 regulatory circuit is so different among clinical isolates? Here, we explore the basis for diversification of this regulatory circuit. Our studies indicate that Wor1 levels modulate the Efg1 regulatory circuit to sculpt natural variation in Efg1-responsive gene expression. Although Wor1 antagonizes Efg1 to direct the white-opaque switch and the white-GUT switch, it collaborates with Efg1 to promote biofilm formation.

## RESULTS

### Promoter region binding of Efg1 in diverse C. albicans clinical isolates.

There is extensive variation in the Efg1-responsive transcriptome among five C. albicans clinical isolates (13). These isolates represent five different clades and vary in ability to produce hyphae and biofilm (11, 13). Genome sequence analysis by Hirakawa et al. (11) indicated that the average genome-wide nucleotide diversity between any two isolates is ~0.37%. To increase confidence in gene expression differences among these isolates, we repeated triplicate RNA-seq assays for the five clinical isolates and *efg1*Δ/Δ derivatives after hyphal induction for 4 h in RPMI plus fetal bovine serum (RPMI+FBS) at 37°C (see Data Set S1 in the supplemental material, all genes). Analysis of the pooled data sets defined a set of 200 genes that presented significant Efg1-responsive RNA levels in all strains (>2-fold change between *efg1*Δ/Δ and WT; adjusted *P* < 0.05). We refer to these 200 genes as core Efg1-responsive genes (see Data Set S1, down and up data). Data set comparisons also identified 1,051 genes with Efg1-responsive RNA levels in only one or a few strains. We refer to these 1,051 genes as strain-limited Efg1-responsive genes (see Data Set S1, down and up); they represent natural variation in the Efg1 regulatory circuit. We sought to determine the basis for this natural variation.

10.1128/mbio.01937-22.2 DATA SET S1 Efg1 RNA-seq data. Expression ratios are provided for *efg1*Δ/Δ versus wild-type strains in each clinical isolate background. Source data include 3 biological replicates per strain from Huang et al. (13) and 3 biological replicates per strain from the current study. Download Data Set S1, XLSX file, 3.3 MB. Copyright © 2022 Do et al. 2022 Do et al. https://creativecommons.org/licenses/by/4.0/ This content is distributed under the terms of the Creative Commons Attribution 4.0 International license.

**FIG 1:**
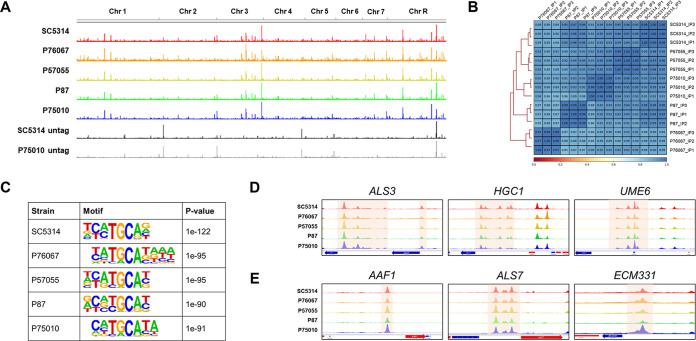
Preservation of Efg1 genomic binding sites among C. albicans clinical isolates. (A) Overview of genomic Efg1 binding sites in 5 clinical isolates. Each peak represents DNA enrichment from ChIP-seq. Efg1-HA strains in clinical isolates SC5314, P76067, P57055, P87, and P75010 and untagged strains SC5314 and P75010 were visualized using IGV v2.11. The ChIP-seq data shown were derived from three independent biological replicates (*n* = 3). (B) Pearson correlation coefficients between ChIP-seq samples in each strain background were measured using multiBamSummary (Galaxy version 3.3.2.0.0) with a 500-bp bin size. The heatmap was generated using plotCorrelation (Galaxy version 3.3.2.0.0). (C) Results of the *de novo* binding motif analysis for each strain, performed in 200-bp regions centered on the peak summits, using HOMER v4.11. (D and E) DNA enrichment from Efg1 ChIP-seq at the promoter regions of target genes, showing Efg1 binding peaks upstream of core direct Efg1 targets *ALS3*, *HGC1*, and *UME6* (D) and upstream of strain-limited direct Efg1 targets *AAF1*, *ALS7*, and *ECM331* (E).

One possible explanation for variation in Efg1-responsive genes is that Efg1 binds to distinct genomic loci in each strain. Differences in the binding spectrum would lead to different consequences for loss of Efg1. To identify loci to which Efg1 binds, we conducted chromatin immunoprecipitation sequencing (ChIP-seq) analysis of epitope-tagged Efg1-hemagglutinin (Efg1-HA) in each clinical isolate background. Samples were prepared after hyphal induction for 4 h in RPMI+FBS at 37°C. Efg1-HA presented consistent binding loci in all five clinical isolates (Fig. 1A; see also Data Set S2). Pearson's coefficients for 500-bp bins across the genome indicated that samples correlated strongly, with *R* values ranging from 0.85 to 1.00 (Fig. 1B). Computed Efg1 binding motifs for each clinical isolate showed that bound regions were associated with the canonical Efg1 binding motif, 5′-TGCAT-3′ (Fig. 1C). These results argue that Efg1-target binding is largely uniform among the five C. albicans isolates.

10.1128/mbio.01937-22.3 DATA SET S2 Efg1-HA binding peaks. Genomic coordinates of Efg1-HA ChIP-seq peaks as defined by MSPC are listed. Raw data are available through NCBI SRA accession number PRJNA849610. Download Data Set S2, XLSX file, 0.8 MB. Copyright © 2022 Do et al. 2022 Do et al. https://creativecommons.org/licenses/by/4.0/ This content is distributed under the terms of the Creative Commons Attribution 4.0 International license.

### Efg1 core network of direct target genes.

**FIG 2:**
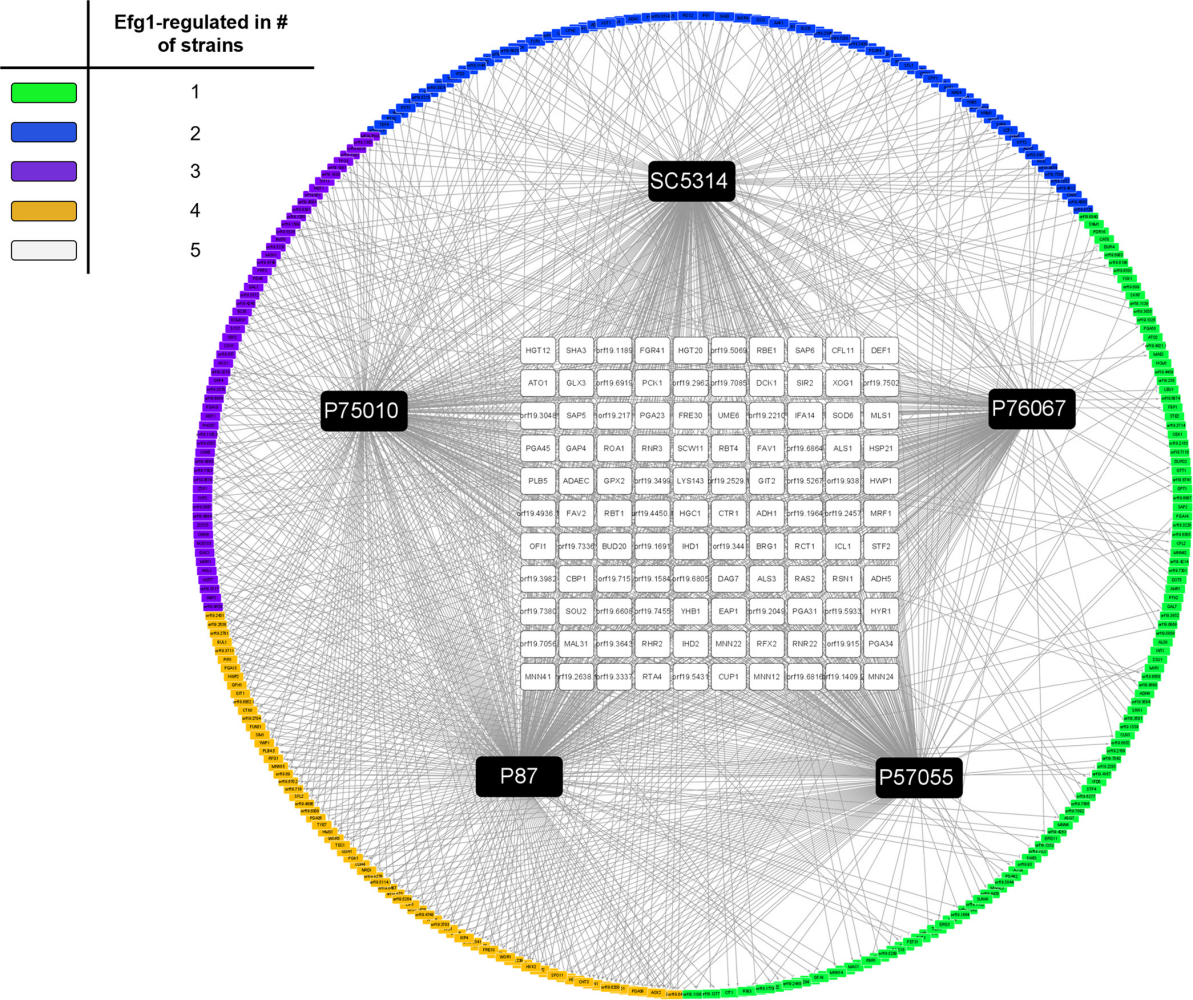
Efg1 direct target genes in C. albicans clinical isolates. In this network diagram, black boxes represent Efg1 for each strain and are labeled with strain names. Nodes represent Efg1 direct target genes, and node colors reflect the number of isolates that displayed Efg1-responsive gene regulation as follows: white, 5; yellow, 4; purple, 3; blue, 2; green, 1. Core direct target genes are represented by the white boxes; strain-limited direct target genes are represented by the yellow, purple, blue, and green boxes.

**FIG 3:**
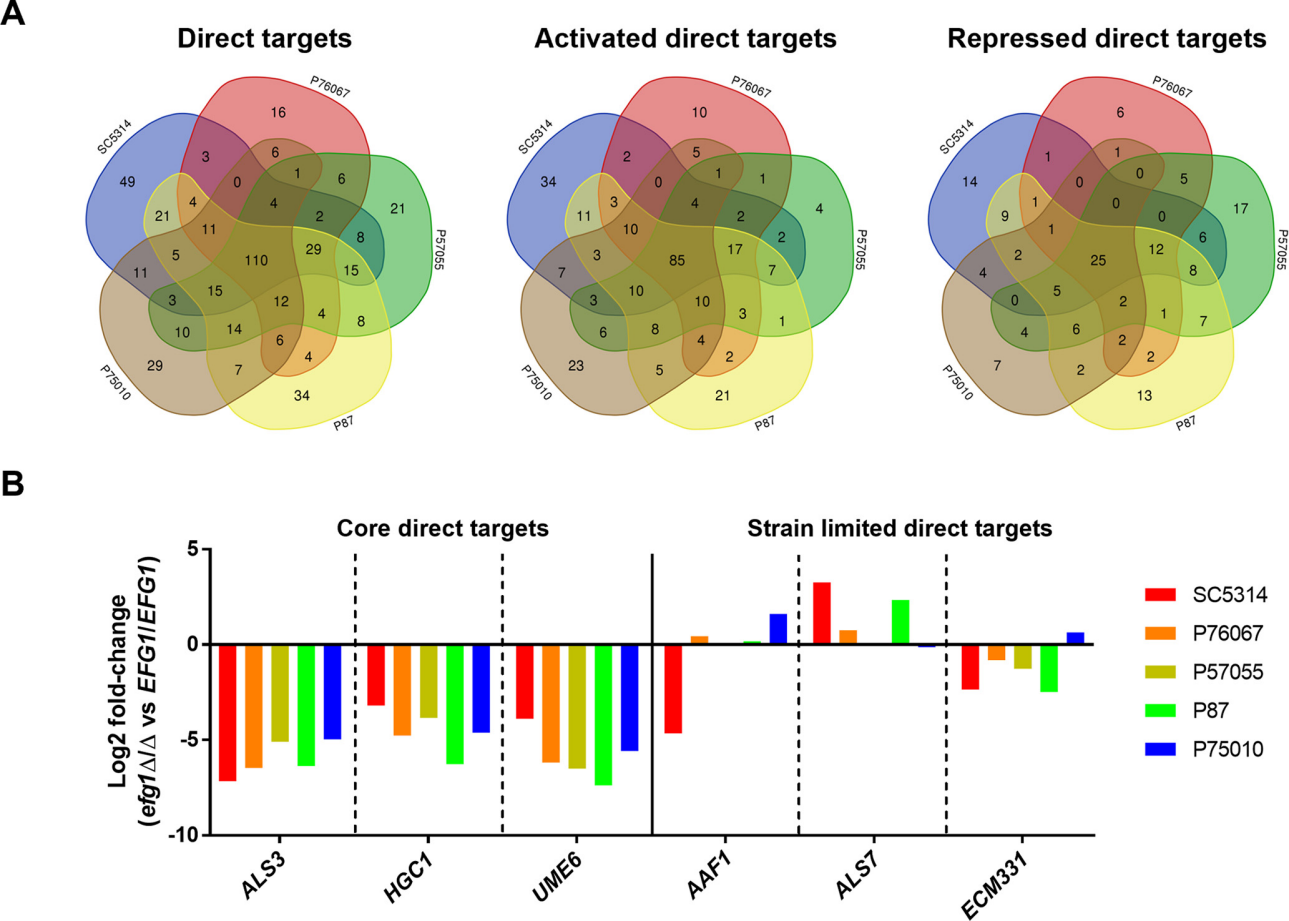
Regulatory diversity of Efg1 direct target genes. (A) Venn diagrams summarizing regulation of Efg1 direct target genes in each clinical isolate. Numbers in the Venn diagrams indicate Efg1 direct targets that were significantly differentially expressed between the *efg1*Δ/Δ mutant and wild type in each background. Note that oppositely regulated genes, i.e., those that were activated by Efg1 in one strain and repressed by Efg1 in another, were counted twice. (B) Graph indicating log_2_ fold change for each Efg1 direct target gene displayed in Fig. 1C and D. Data represent means of *n* = 6 biologically independent samples. Source data are provided in Table S1 in the supplemental material under differential expression.

We merged RNA-seq and ChIP-seq data to identify genes that were both differentially expressed in *efg1*Δ/Δ mutants and bound by Efg1. This analysis showed that 218 to 299 genes were regulated directly by Efg1 in each clinical isolate, and 110 genes were regulated directly by Efg1 in all five clinical isolates (Fig. 2; see also Table S1, Efg1 direct target gene ID and core direct Efg1 targets). The 110 genes, which we call core direct Efg1 targets, included 85 Efg1-activated genes and 25 Efg1-repressed genes (Fig. 3A). Core direct targets were enriched for functions related to adhesion and biofilm formation (adhesion of symbiont to host, *P* = 1.48e−06; single-species biofilm formation, *P* = 8.87e−06), in keeping with the observation that Efg1 is required for biofilm formation in all clinical isolates tested (13).

10.1128/mbio.01937-22.5 TABLE S1 Efg1 direct target information. Download Table S1, XLSX file, 0.2 MB. Copyright © 2022 Do et al. 2022 Do et al. https://creativecommons.org/licenses/by/4.0/ This content is distributed under the terms of the Creative Commons Attribution 4.0 International license.

Examination of binding peaks surrounding specific genes indicated that Efg1 binding differences were not the cause of regulatory differences among strains. For example, core direct targets *UME6*, *ALS3*, and *HGC1* were under positive control by Efg1 in all strains examined (Fig. 3B; see also Table S1, core direct Efg1 targets). The 5′ regions of these genes were uniformly bound by Efg1 (Fig. 1D). In contrast, *AAF1*, *ALS7*, and *ECM331* were regulated by Efg1 in some strains and not in others (Fig. 3B; see also Table S1, differential expression). The 5′ regions of these genes were also uniformly bound by Efg1 (Fig. 1E). Therefore, differences in Efg1-responsive expression of genes cannot be explained by differences in Efg1-promoter region binding.

### Strain-limited promoter response to Efg1.

**FIG 4:**
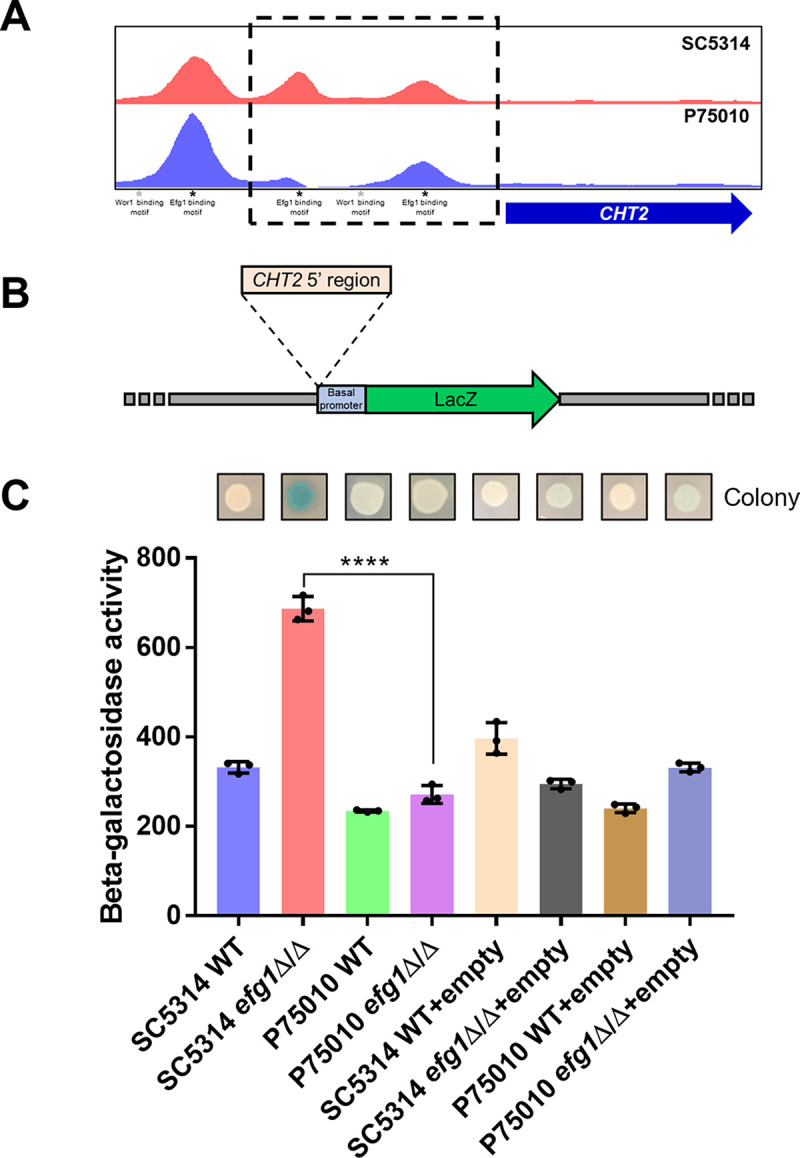
Efg1-responsive regulation of the *CHT2* upstream region in strains SC5314 and P75010. (A) Efg1 binding peaks in the *CHT2* 5′ region. Locations of binding peaks visualized with IGV-viewer for SC5314 and P75010 are shown, along with locations of binding motifs for Efg1 and Wor1. The boxed region was used as the *CHT2* 5′ region with the *LacZ* reporter. (B) Diagram of the *LacZ* reporter gene driven by the *CHT2* 5′ region. The empty vector contains the basal promoter region of *ADH1* followed by the *LacZ* coding region. The *CHT2* 5′ region was inserted upstream of the basal promoter to create the reporter gene. (C) β-Galactosidase activity. Colonies grown on an RPMI with 10% FBS plate at 37°C for 24 h were stained for β-galactosidase activity and photographed. β-Galactosidase activity was quantified from planktonic cells grown in RPMI +10% FBS at 37°C for 24 h. Soluble extracts were incubated with ortho-nitrophenyl-d-galactopyranoside (ONPG), and hydrolyzed ONPG was measured. Statistical significance was determined with an unpaired, two-tailed Student's *t* test. ****, *P* < 0.0001. Data represent means ± standard deviations of *n* = 3 biologically independent samples.

To determine whether strain differences in Efg1-responsive gene expression reflect sequence differences in promoter or regulatory regions, we focused on the *CHT2* gene. Its RNA levels were repressed by Efg1 in strain SC5314 but activated slightly by Efg1 in strain P75010 (see Table S1, differential expression). To assay activity of the same promoter sequence in both strains, we fused a 1,743-bp fragment from the SC5314 *CHT2* 5′ region to a *lacZ* reporter gene and introduced the construct into each wild type (WT) and its derived *efg1*Δ/Δ mutant (Fig. 4A and B). Measurement of β-galactosidase indicated that the reporter gene was repressed by Efg1 in strain SC5314 and unaffected by Efg1 in strain P75010 (Fig. 4C). These assays were conducted after hyphal induction for 24 h in RPMI+FBS at 37°C, allowing time for β-galactosidase to accumulate. Repression required the *CHT2* promoter region (Fig. 4C). These results showed that identical C. albicans promoter sequences respond differently to Efg1 in these two strain backgrounds.

### Sculpting of Efg1 transcriptional target space by partner TFs.

**FIG 5:**
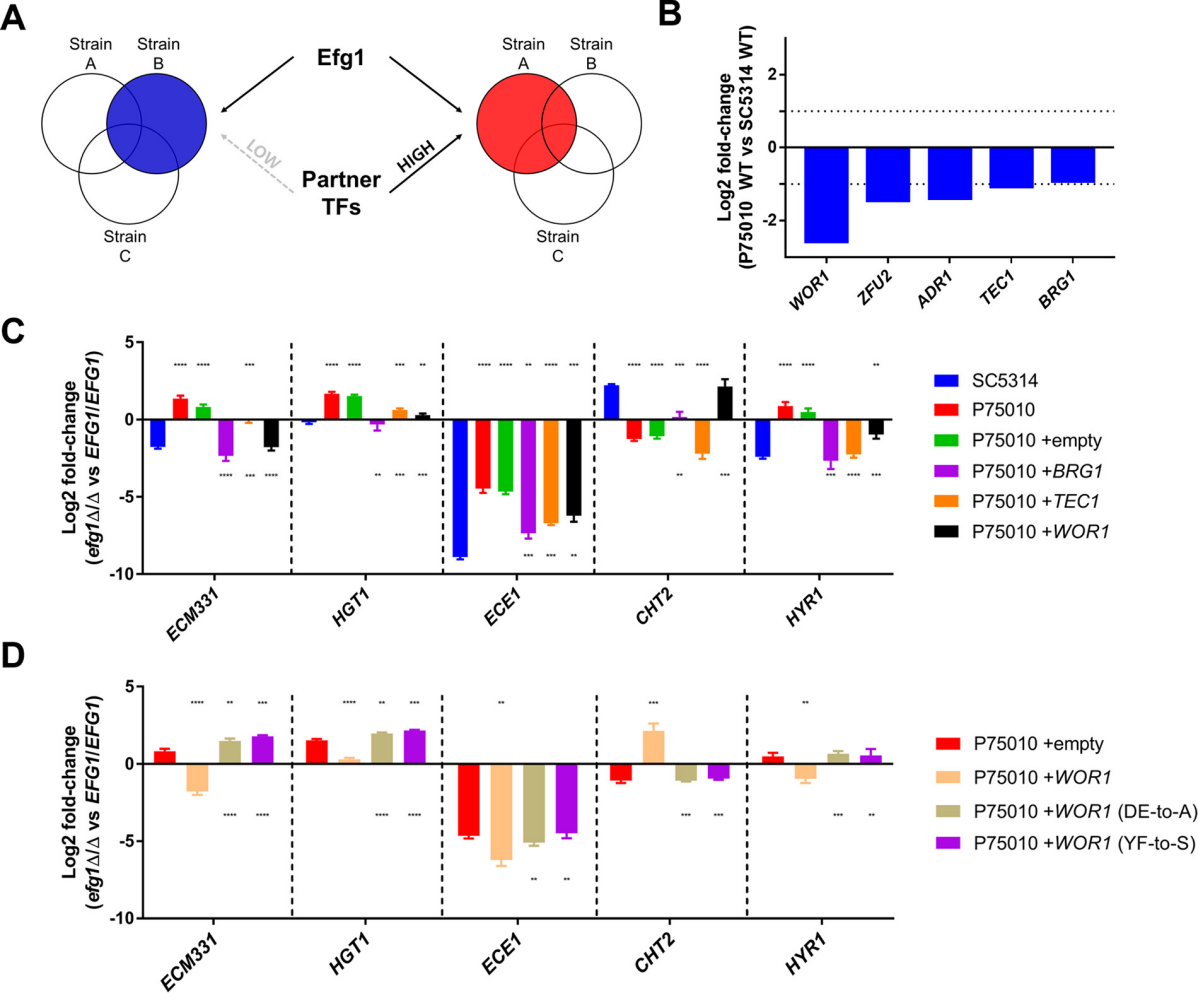
Sculpting of the Efg1 regulatory network by ectopic expression of Efg1 partner TFs. (A) Diagram depicting the hypothesis that natural variation of Efg1 regulatory networks reflects differences in abundance of partner regulators in each clinical isolate. (B) Graph indicating log_2_ fold change for each TF gene in P75010 compared to SC5314. Data represent means of *n* = 6 biologically independent samples. Values were calculated by using Deseq2 (Benjamini-Hochberg adjusted *P* values of <0.05) (see Table S2). (C and D) Graphs indicating log_2_ fold changes in RNA levels for *ECM331*, *HGT1*, *ECE1*, *CHT2*, and *HYR1.* Strains were grown in RPMI +10% FBS at 37°C for 4 h, and RNA was extracted for NanoString analysis. Gene expression ratios were calculated using mRNA counts of three independent biological samples between mutant and wild type, and statistical significance was determined by the Benjamini-Hochberg step-up procedure; false-discovery rate = 0.1. See Table S3, ectopic strains *efg1* versus *EFG1*, for numeric data. Statistical significance between gene expression ratios was determined with an unpaired, two-tailed Student's *t* test. **, *P* < 0.01; ***, *P* < 0.001; ****, *P* < 0.0001. In panel C, asterisks above the bars indicate statistical significance compared with SC5314 and asterisk marks below the bars indicate statistical significance compared with P75010. In panel D, asterisks above the bars indicate statistical significance compared with P75010+empty, and asterisk marks below the bars indicate statistical significance compared with P75010+*WOR1*.

To explore the basis for strain variation in Efg1-responsive gene expression, we focused on the two strains SC5314 and P75010. SC5314 is the type strain for C. albicans and was isolated from a patient with a generalized *Candida* infection (http://www.candidagenome.org/Strains.shtml). P75010 is a bloodstream isolate (14). We hypothesized that differences in expression or activity of TFs that are functionally related to Efg1 may explain natural variation in the Efg1 regulatory response (Fig. 5A). TF genes that were expressed at higher levels in SC5314 than in P75010 (Fig. 5B; see also Table S2) included *WOR1* (regulator of white-opaque switching), *ZFU2* (regulator of cell adherence), *ADR1* (regulator of carbon metabolism), and *TEC1* and *BRG1* (regulators of biofilm and hyphal formation). We increased dosages of individual TF genes in P75010 to test whether their increased expression altered Efg1-responsive gene expression.

10.1128/mbio.01937-22.6 TABLE S2 Differentially expressed TF genes in P75010. Download Table S2, XLSX file, 0.02 MB. Copyright © 2022 Do et al. 2022 Do et al. https://creativecommons.org/licenses/by/4.0/ This content is distributed under the terms of the Creative Commons Attribution 4.0 International license.

10.1128/mbio.01937-22.7 TABLE S3 Gene expression data for ectopic expression strains. Download Table S3, XLSX file, 0.1 MB. Copyright © 2022 Do et al. 2022 Do et al. https://creativecommons.org/licenses/by/4.0/ This content is distributed under the terms of the Creative Commons Attribution 4.0 International license.

Each TF gene, PCR-amplified from strain P75010, was integrated into the genome in P75010 *EFG1*+/+ and *efg1*Δ/Δ strains. Integration was targeted to the *MDR1* locus. (An *mdr1*Δ/Δ mutation lacking an inserted TF gene did not alter gene regulation in our assays [Fig. 5C, P75010+empty; see also Table S3, ectopic strains *efg1* versus *EFG1*.]) Gene expression was assayed after hyphal induction for 4 h in RPMI+FBS at 37°C (Fig. 5C; see also Table S3). Dosage increases yielded 2- to 10-fold increased TF gene expression (see Table S3, WOR1-BRG1-TEC1 relationship). Increased dosage of the known biofilm and hyphal regulatory gene *BRG1* in strain P75010 caused Efg1-responsive regulation of *ECM331*, *HGT1*, *ECE1*, and *HYR1* to resemble more closely that in strain SC5314. Increased dosage of the biofilm and hyphal regulatory gene *TEC1* had similar effects on *ECE1* and *HYR1*. Increased dosage of *ZFU2* and *ADR1* had little effect (see Table S3, ADR1-ZFU2 data). Increased dosage of *WOR1* in P75010 also caused Efg1-responsive regulation to resemble that in SC5314 (Fig. 5C; see also Table S3, ectopic strains *efg1* versus *EFG1*). For example, *ECM331* and *HYR1* were under negative control by Efg1 in P75010 but under positive control in SC5314 and the increased-dosage strain, P75010*+WOR1.* Conversely, *CHT2* was under positive control by Efg1 in P75010 but under negative control in SC5314 and P75010*+WOR1.* Although the range of genes assayed was limited, increased dosage of *WOR1*, *BRG1*, or *TEC1* in strain P75010 caused features of Efg1-dependent gene regulation to resemble those in strain SC5314.

**FIG 6:**
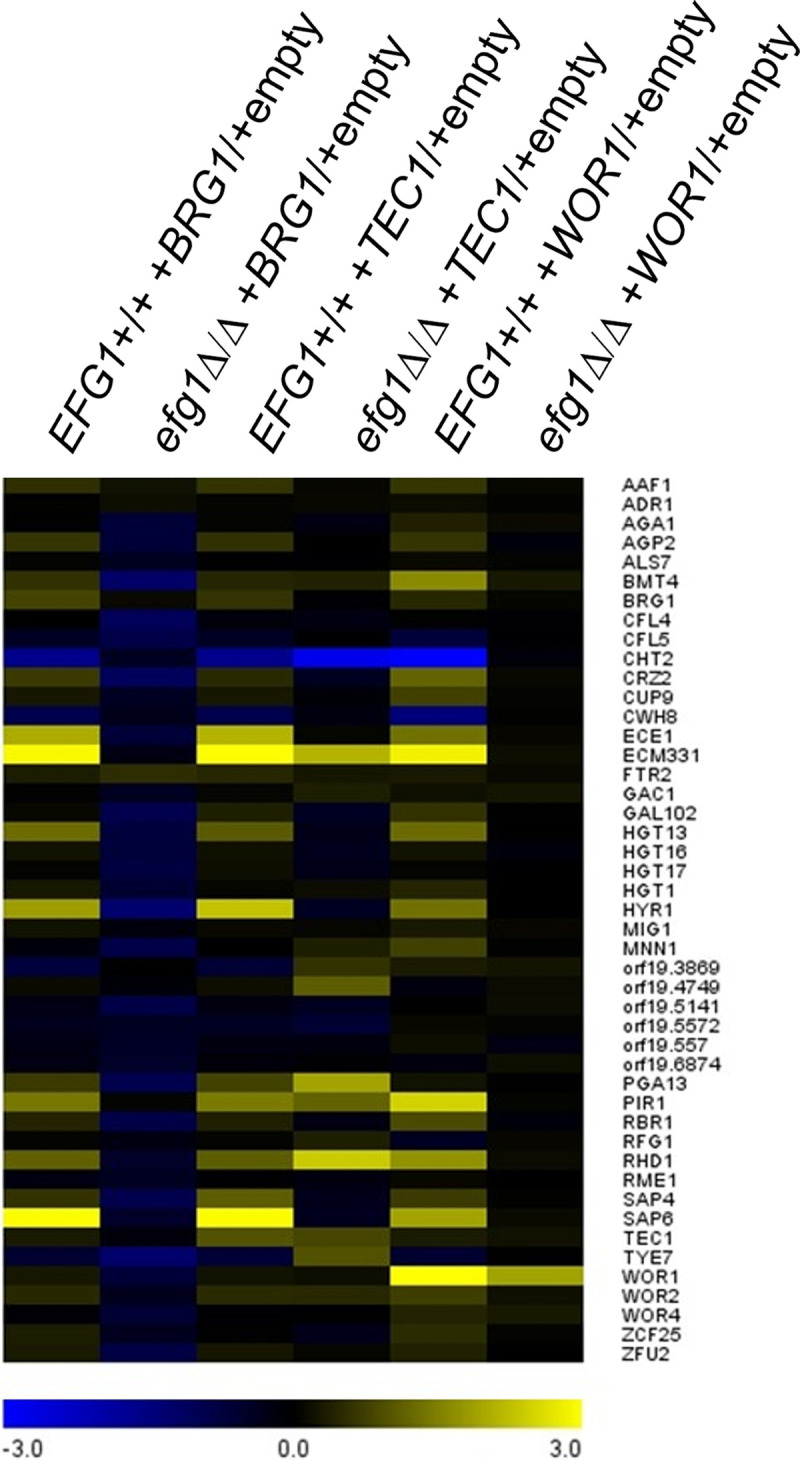
Dependence of partner TF gene expression impact on Efg1. P75010-derived *EFG1+/+* (wild type) and *efg1*Δ/Δ strains with increased dosages of *BRG1*, *TEC1*, or *WOR1* were compared to empty vector controls by nanostring analysis of target gene RNA levels. Results are presented as a heatmap. See Table S3, normalized counts, for NanoString platform data.

To determine whether the gene expression effects of increased TF gene dosage depend upon Efg1, we compared gene dosage impact in P75010 *EFG1*+/+ and *efg1*Δ/Δ backgrounds (Fig. 6; see also Table S3, ectopic strains versus empty). Increased *BRG1* dosage had significant effects on 10 genes (adjusted *P* < 0.05; excluding *BRG1* itself) in the *EFG1*+/+ background, and those effects were abolished in the *efg1*Δ/Δ background. Therefore, effects of increased *BRG1* dosage on these genes depend upon Efg1. Increased *TEC1* dosage had significant effects on 15 genes (excluding *TEC1*) in an *EFG1*+/+ background, and only some of those effects were abolished in an *efg1*Δ/Δ background. Therefore, effects of increased *TEC1* dosage are partially independent of Efg1. Increased *WOR1* dosage had significant effects on 10 genes (excluding *WOR1*) in the *EFG1*+/+ background, and those effects were abolished in the *efg1*Δ/Δ background. Therefore, effects of increased *WOR1* dosage on these genes depend upon Efg1.

### Positive control of biofilm formation by Wor1 in P75010.

**FIG 7:**
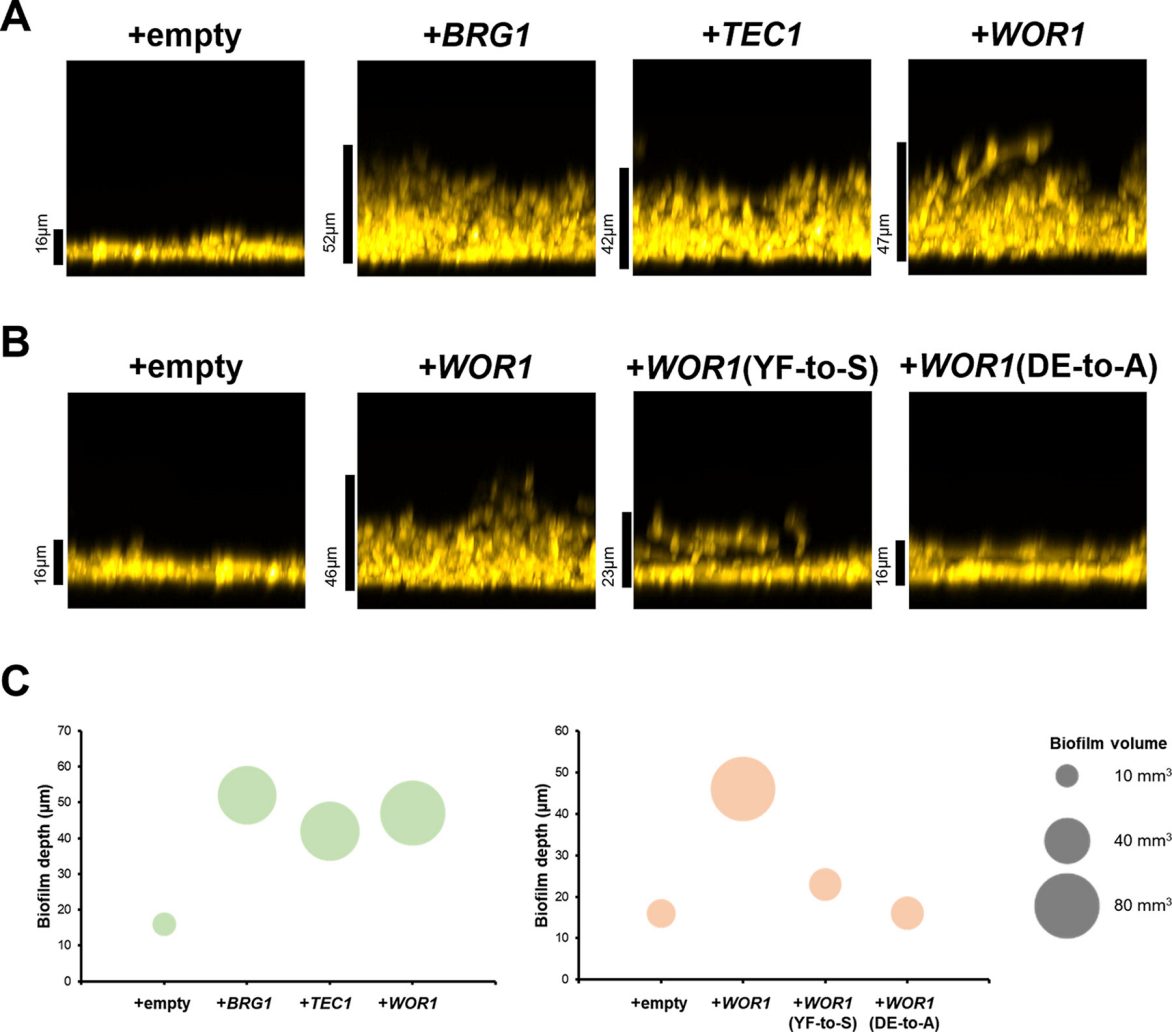
Stimulation of biofilm formation by partner TFs. (A and B) Biofilm side-view projections. P75010 wild type and derivatives were assayed for biofilm formation. Strains were grown in RPMI with 10% FBS for 24 h at 37°C in a 96-well plate. Fixed biofilms were stained with calcofluor white and imaged using a Keyence BZ-X800E fluorescence microscope. Scale bars indicate depth of the corresponding biofilm. (C) Biofilm volume, measured with Image J and presented in a bubble plot. The *y* axis indicates the biofilm depth (in micrometers), and bubble size represents the biofilm volume (in cubic millimiters). A volume scale is shown on the right.

We hypothesized that the gene expression impact of increased *WOR1*, *BRG1*, or *TEC1* dosage may translate into biological phenotype. Strain SC5314 is a stronger biofilm former than P75010 (13). We observed that increased dosage of *WOR1*, *BRG1*, or *TEC1* in P75010 drove greater biofilm production (Fig. 7A), based upon either depth or volume (Fig. 7C). We concluded that differences in *WOR1*, *BRG1*, or *TEC1* expression levels between strains can account for differences in both biofilm formation ability and Efg1-responsive gene expression.

The Efg1-Wor1 physical interaction *in vitro* depends upon the Wor1 PrLD, a region that is required for Wor1 function in the white-opaque switch (10). We hypothesized that the Efg1-Wor1 functional interaction in gene expression and biofilm formation may also be PrLD dependent. In our increased dosage assay, PrLD-disrupted *WOR1* mutants (DE-to-A or YF-to-S) had much less impact than wild-type *WOR1* in Efg1-responsive regulation of *ECM331*, *HGT1*, *ECE1*, *CHT2*, or *HYR1* (Fig. 5D; see also Table S3, ectopic strains *efg1* versus *EFG1*) or biofilm production (Fig. 7B and C). Therefore, integrity of the Wor1 PrLD is required for Wor1 to impact both Efg1-responsive gene expression and biofilm formation.

If Wor1 promotes biofilm formation through interaction with Efg1, then an *efg1*Δ/Δ mutation should abrogate the stimulation of biofilm formation by increased *WOR1* dosage. We observed that an *efg1*Δ/Δ mutation blocked biofilm formation in strain P75010 regardless of increased *WOR1* dosage (see Fig. S1). Therefore, the impact of *WOR1* dosage on biofilm formation, like its impact on Efg1-responsive gene expression, depends upon Efg1.

10.1128/mbio.01937-22.8 FIG S1 Impact of increased *WOR1* dosage. Biofilm formation by P75010 *efg1*Δ/Δ and *efg1*Δ/Δ+*WOR1* strains. (A) Strains were grown in RPMI plus 10% FBS for 24 h at 37°C in a 96-well plate. Fixed biofilms were stained with calcofluor white and imaged using a Keyence BZ-X800E fluorescence microscope. Side-view projections are shown. (B) Biofilm formation by each strain was determined in a tetrazolium salt (XTT) reduction assay. Strains were grown in RPMI with 10% FBS for 24 h at 37°C in a 96-well plate, and then biofilms were incubated with phosphate-buffered saline containing XTT-menadione at 37°C for 30 min in the dark. Reduced XTT was determined by using a plate reader at 492 nm. Statistical significance was determined with an unpaired, two-tailed Student’s *t* test. (C) Venn diagram depicting intersection between the P75010 Efg1 direct target genes and the differentially expressed genes in P75010+*WOR1* strain compared to the P75010+empty strain. Source gene expression data are provided in Data Set S3. Download FIG S1, TIF file, 0.4 MB. Copyright © 2022 Do et al. 2022 Do et al. https://creativecommons.org/licenses/by/4.0/ This content is distributed under the terms of the Creative Commons Attribution 4.0 International license.

10.1128/mbio.01937-22.8 FIG S2 Efg1-HA proteins in *C. albicans* clinical isolates. (A) Construction of homozygous Efg1-HA-tagged derivatives of each clinical isolate. (B) Wild-type and Efg1-HA tagged derivatives for each strain background were assayed for filamentation under planktonic growth conditions. Strains were grown in RPMI with 10% FBS at 37°C for 4 h. Cells were fixed with 4% formaldehyde and then stained with calcofluor white for fluorescence microscopy. White scale bars, 10 μm. (C) Western blot analysis was performed using SC5314 wild-type and Efg1-HA-tagged strains. Strains were grown in RPMI + 10% FBS at 37°C for 4 h with shaking, and then soluble proteins were extracted. The asterisk indicates a nonspecific band. The Ponceau S-stained membrane was used as a loading control. (D) Western blot analysis of Efg1-HA-tagged derivatives of each clinical isolate. Strains were grown in RPMI plus 10% FBS at 37°C for the indicated incubation times with shaking, and then soluble proteins were extracted. The Ponceau S-stained membrane was used as a loading control. (E) Western blot analysis was performed using total soluble proteins from Sc5314 Efg1-HA cells grown in RPMI plus 10% FBS at 37°C. Growth times are indicated in hours. For dephosphorylation of Efg1-HA, the cell lysate from SC5314 Efg1-HA was treated with lambda phosphatase at 37°C for 1 h prior to processing for electrophoresis. The Ponceau S-stained membrane was used as a loading control. Download FIG S2, TIF file, 1.6 MB. Copyright © 2022 Do et al. 2022 Do et al. https://creativecommons.org/licenses/by/4.0/ This content is distributed under the terms of the Creative Commons Attribution 4.0 International license.

10.1128/mbio.01937-22.4 DATA SET S3 *WOR1* RNA-seq data. Expression ratios are provided for P75010+*WOR1* and P75010+empty vector strains. Source data include 3 biological replicates per strain from the current study. Download Data Set S3, XLSX file, 0.4 MB. Copyright © 2022 Do et al. 2022 Do et al. https://creativecommons.org/licenses/by/4.0/ This content is distributed under the terms of the Creative Commons Attribution 4.0 International license.

In order to understand the scope of the impact of *WOR1* gene expression, we conducted RNA-seq analysis on P75010+empty vector and P75010+*WOR1* strains. Increased *WOR1* dosage altered expression of 396 genes (293 upregulated, 103 downregulated; fold change, >2; adjusted *P* < 0.05 [see Data Set S3]). Upregulated genes were enriched for adhesion functions and included many genes associated with biofilm or hyphal growth (*ALS1*, *HYR1*, *SAP4-SAP5-SAP6*, and *UME6*). The 396 genes with significantly altered expression were enriched for Efg1-bound genes (*P* < 10^−5^, Fisher’s exact test [see Fig. S1]). *EFG1* RNA levels were not significantly affected (see Data Set S3). Therefore, increased *WOR1* dosage has a narrow effect on gene expression under these growth conditions. Based on *WOR1* dosage effects on gene expression and biofilm formation, our findings support the model that Wor1 acts in part through Efg1 to modulate gene expression.

## DISCUSSION

**FIG 8:**
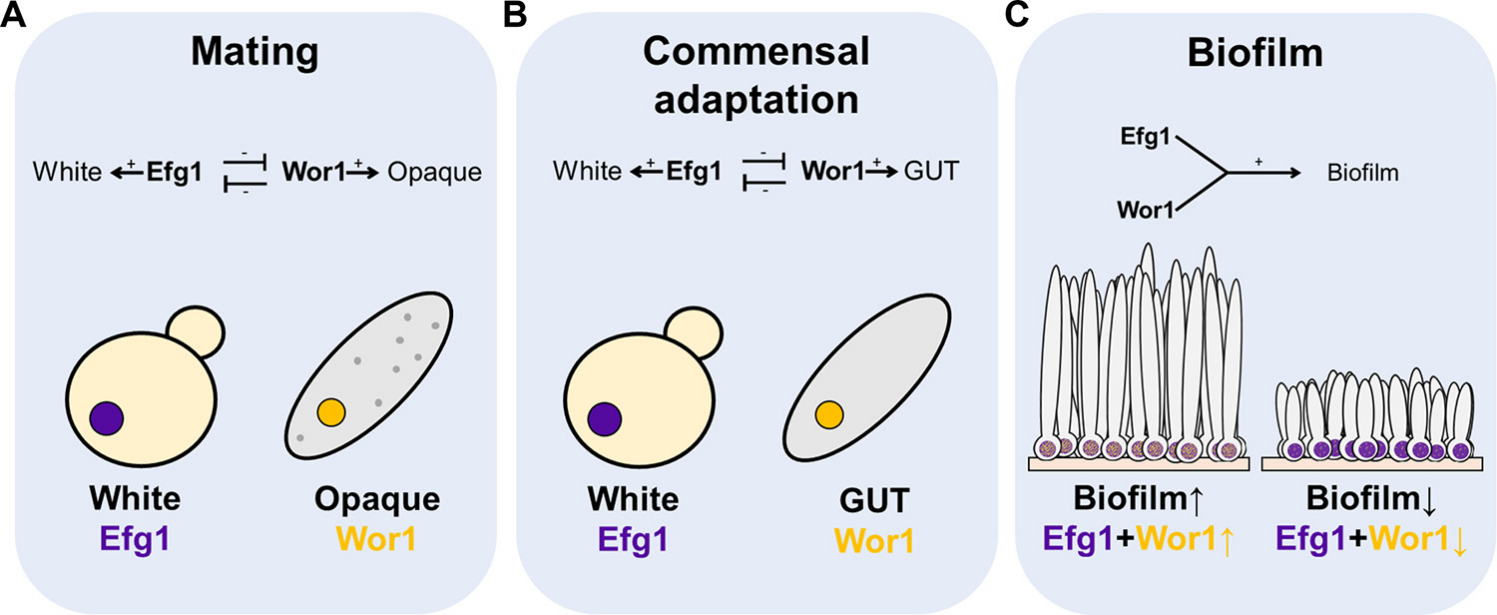
Collaboration between antagonistic cell type regulators in the biofilm/hyphal gene expression network. (A and B) Cell type determination. Each cell morphotype—white, GUT, and opaque—has a distinct gene expression level of master regulators. High levels of Efg1 repress *WOR1* and favor the white cell type, while high levels of Wor1 repress *EFG1* and favor the GUT or opaque states. (C) Biofilm production. Under biofilm conditions, both Efg1 and Wor1 are present and cooperate to drive biofilm formation. In our study, efficient biofilm-forming strains had higher *WOR1*:*EFG1* RNA level ratios (SC5314, 0.037; P75010*+WOR1*, 0.22) than the inefficient biofilm-forming strain (P75010, 0.005), as calculated from RNA-seq data (see Data sets S1 and S3). We propose that differences in balance of Efg1 and Wor1 can account for natural variation in biofilm production.

Isolates of C. albicans vary for a range of properties connected to biofilm, hyphae, and the regulation of hypha-associated genes. In this report, we sought to determine the basis for natural variation in gene expression responses, with a focus on the well-characterized biofilm and hyphal regulator Efg1. Our findings revealed that the genomic distribution of Efg1 binding regions is essentially uniform among strains, whereas the gene expression impact of bound Efg1 is not. We observed that modest differences in expression of TF genes with some functional connection to Efg1 can have substantial impact on Efg1-responsive gene expression; they can even determine whether genes are under positive or negative Efg1 control. This partner TF analysis revealed that Wor1, a white-opaque regulator known to antagonize Efg1 in cell type switching and commensalism, has an unexpected positive role in biofilm formation. The positive role depends upon determinants of the Wor1 PrLD, which has been shown to mediate Efg1-Wor1 interactions *in vitro* and in heterologous cells. It is possible that a PrLD-dependent Efg1-Wor1 interaction also functions in biofilm formation (Fig. 8).

### Efg1 core direct target genes.

A group of 110 genes have 5′ regions bound by Efg1 and expression levels that are up- or downregulated by an *efg1*Δ/Δ mutation in all five strains examined. Most of these genes are under positive control by Efg1. This group (see Table S1, core direct Efg1 targets) includes well-characterized determinants of biofilm or hypha formation, such as *ALS1*, *ALS3*, *HGC1*, *HWP1*, and *XOG1*. The uniform regulatory response of these genes aligns well with the uniform biofilm and hyphal defect for *efg1*Δ/Δ mutants among these strains (13). In addition, two Efg1 core direct targets specify the biofilm and hyphal transcriptional regulators Brg1 and Ume6. Thus, the previously described Efg1-Brg1-Ume6 feed-forward loop is preserved among multiple strains, in support of its functional significance (3, 4, 15). Efg1 core direct target genes also include several central metabolic genes, such as *ADH1*, *GLK1*, *HGT6*, and *PCK1*. The products of these genes may help balance competing carbon demands from biofilm matrix synthesis and from energy production and small-molecule synthesis. Still, most of the Efg1 core direct targets lack known or easily rationalized roles in biofilm or hypha formation. This limitation in our understanding may reflect functional redundancy (2, 3, 16–22) or perhaps the limited range of biofilm-related phenotypes that have been assayed in mutant analyses.

### Efg1 strain-limited direct target genes.

Each strain had ~100 to 200 genes whose 5′ regions were bound by Efg1 in all strains but displayed Efg1-responsive expression in only a subset of strains (see Table S1, direct repressed genes and direct activated genes). Strain-limited regulatory responses might be enriched for “noisy” genes (23); we tried to minimize this concern by using data from six biological replicates of WT and *efg1*Δ/Δ derivatives of each strain. We calculate that 51 to 62% of strain-limited direct targets have a variance in reads per kilobase per million values below the mean for our complete RNA-seq data set, compared to 31 to 58% of core direct targets (see Data Set S1, variance summary). Therefore, strain-limited direct targets do not show greater day-to-day variation than core direct targets.

Many strain-limited genes had expression changes that trended in the same direction in all strains but failed to meet our criteria for fold change or statistical significance in one strain. The Efg1-bound genes *SFL2*, *TYE7*, and *YWP1* are examples. Several of these genes impact biofilm and hyphal formation, thus illustrating that strain-limited bound genes may contribute to the *efg1*Δ/Δ mutant phenotype. Even the strain-limited bound genes that showed significant expression changes in only one strain background included some with biofilm- and hypha-related functions, such as *AHR1*, *CLN3*, and *ROB1*. Their expression differences may have contributed to the *efg1*Δ/Δ mutant phenotype in the respective strains as well. However, enrichment for biofilm and hyphal functions is much greater among core direct targets than among strain-limited direct targets.

One class of strain-limited direct targets was activated by Efg1 in some strains and repressed by Efg1 in others (see Table S1, differential expression). Sugar transporter genes *HGT1*, *HGT2*, and *HGT13* were members of this group, as were ferric reductase-like genes *CFL4* and *CFL5* and the chitinase gene *CHT2.* In the case of *CHT2*, our fusion gene analysis (Fig. 4) showed that the Efg1 impact on promoter activity was strain dependent. The existence of such divergent regulatory responses among strains suggests that the net impact of 5′ region-bound Efg1 depends upon the presence of neighboring TFs.

### Wor1 function in the Efg1 regulatory circuit.

What are the determinants of strain-limited regulatory responses? TFs that share target genes with Efg1 are candidates that may modulate activity of 5′ region-bound Efg1. This idea led us to look among TF genes whose RNA levels differed between two strains, SC5314 and P75010. Two candidate TF genes specify known regulators of biofilm- and hypha-related gene expression, Brg1 and Tec1. Increased dosage of *BRG1* in strain P75010, to mimic the naturally elevated *BRG1* expression of SC5314, caused Efg1-responsive regulation of *ECM331*, *HGT1*, *ECE1*, and *HYR1* resembling more closely that in strain SC5314. Increased dosage of *TEC1* had a similar though less pronounced effect. Increased dosage of *BRG1* or *TEC1* also had a functional impact, leading to increased biofilm formation by P75010. Given the many interconnections in the biofilm and hypha regulatory network (24), it seems reasonable that increased expression of known biofilm and hyphal regulators would affect Efg1-target relationships.

This strategy also pointed to an unexpected connection between Wor1 and biofilm and hyphal gene expression. *WOR1* was also expressed at higher levels in SC5314 than in P75010, and increased *WOR1* dosage in P75010 caused SC5314-like phenotypes, including increased biofilm formation and features of Efg1-responsive gene regulation. Wor1 mutant derivatives lacking PrLD integrity, which are known to be defective in interaction with Efg1 (10), as well as an *efg1*Δ/Δ mutation, blocked *WOR1* effects on Efg1-responsive gene expression and biofilm formation. These lines of evidence together argue that Wor1 acts in conjunction with Efg1 under biofilm and hyphal growth conditions.

The findings presented here broaden our view of the relationship between Wor1 and Efg1 (Fig. 8). White-opaque and white-GUT cell type switching reflect antagonism between Efg1 and Wor1 (1, 5–7, 9). The white state is at one extreme, in which Efg1 expression and function predominate; the opaque or GUT state is at the other extreme, in which Wor1 expression and function predominate (1, 5–7, 9). However, our analysis of natural variation indicated that a more delicate balance between Wor1 and Efg1 activities had an impact on the capacity to form biofilm and the regulation of Efg1 target genes. The impact of *WOR1* in this context supports a new feature of Wor1 function: that Wor1 acts in collaboration with Efg1 in the biofilm and hyphal regulatory network.

What is the nature of the Wor1-Efg1 collaboration? What mechanism enables Wor1 levels to affect Efg1-responsive gene expression? One model is that Wor1 interacts directly with Efg1 to modify its activity. Most Efg1 direct targets are upregulated in P75010*+WOR1* compared to P75010+empty vector, suggesting that a hypothetical Wor1-Efg1 complex may have greater activation ability than Efg1 alone. A second model is that Wor1 acts more indirectly to promote expression or activity of known biofilm and hyphal activators that share targets with Efg1. The best candidates from our data are Brg1 and to a lesser extent Tec1, based on the parallels in dosage effects. Specifically, increased dosage of *BRG1*, *TEC1*, or *WOR1* affected an overlapping set of genes (Fig. 6) and increased biofilm formation (Fig. 7A) in strain P75010. *BRG1* and *TEC1* RNA levels were elevated ~1.5-fold in P75010*+WOR1* compared to P75010+empty vector, increases that make this model seem possible if perhaps not persuasive. The mechanism through which Efg1 and Wor1 act to promote biofilm and hyphal gene expression and biofilm formation remains uncertain at this time.

### Natural variation-driven functional discovery.

Our findings here emphasize the value of natural strain variation for functional genetic analysis in C. albicans. Natural variation has been exploited previously to define mechanisms of drug resistance (25, 26), genome evolution (27), and virulence determinants (28). However, the lack of a complete sexual cycle precludes genome-wide association studies and related mapping approaches, and the high level of sequence variation among strains makes candidate genes too numerous to explore efficiently. The type strain SC5314 has been a reliable starting point to define genetic determinants of diverse biological processes with reverse-genetics manipulations. However, the features that make it an excellent workhorse, such as strong biofilm formation and a high level of virulence in diverse infection models, may reflect genetic adaptations that amplify activities of some circuits and minimize the impacts of others. Other clinical isolates, with a different selection of amplified and minimized circuits, offer a sensitive context to reveal new gene functions and functional relationships.

## MATERIALS AND METHODS

### Strains and media.

Strains used in this study were maintained in 15% glycerol frozen stocks at −80°C. Prior to use, cells were routinely grown on YPD agar plates (2% dextrose, 2% Bacto peptone, 1% yeast extract, 2% Bacto agar) overnight at 30°C and then cultured in liquid YPD medium overnight at 30°C with shaking. Transformants were selected on YPD plus 400 μg/mL nourseothricin (clonNAT; Gold Biotechnology) or complete synthetic medium (2% dextrose, 1.7% Difco yeast nitrogen base with ammonium sulfate and auxotrophic supplements). All strains used in this study are listed in Text S1 in the supplemental material.

10.1128/mbio.01937-22.10 TEXT S1 Detailed information on our materials and methods, strains, plasmids, and primer sequences. Download Text S1, DOCX file, 0.1 MB. Copyright © 2022 Do et al. 2022 Do et al. https://creativecommons.org/licenses/by/4.0/ This content is distributed under the terms of the Creative Commons Attribution 4.0 International license.

### Plasmid construction.

Plasmid construction employed routine methods and is detailed in Text S1. Primers and plasmids are listed in Text S1.

### Strain construction.

To manipulate the C. albicans genome, the transient CRISPR-Cas9 system was employed as previously described in detail (29). Generally, the Cas9 cassette was amplified from the plasmid pV1093, and each single guide RNA (sgRNA) cassette was generated by using split-joint PCR with sgRNA/F YFG1 and SNR52/R YFG1 as previously described in detail (13, 29). Additional details about strain construction are provided in Text S1.

### Filamentation assay.

To assay hyphal formation in C. albicans strains, cell culture and fixation were performed according to previously published methods (13), as detailed in Text S1.

### NanoString analysis.

Cells grown in 5 mL YPD overnight at 30°C were washed and then cultured in 25 mL of RPMI with 10% FBS for 4 h at 37°C. Cell harvest and RNA extraction were performed as previously described (30). NanoString analysis was performed as previously described (31). Briefly, total 15 ng of RNA from each strain was used for hybridization with code set and capture probe for 16 h at 65°C. Gene expression levels were measured by an nCounter SPRINT Profiler, and data were normalized with 5 genes (*ARP3*, *CDC28*, *FKH2*, *GIN4*, and *TUP1*) using NanoString nSolver v4.0.

### RNA-seq analysis.

RNA preparation and RNA-seq analysis were performed as described in Text S1 and in reference 13.

### Chromatin immunoprecipitation and ChIP-seq library preparation.

Chromatin immunoprecipitation and ChIP-seq library preparation are detailed in Text S1 and were based on methods described in reference 32.

### Bioinformatic analysis.

Raw Illumina fastq data from all strains and samples were aligned to the C. albicans genome release 21 using bowtie2 (v 2.1.0; default options), as detailed in Text S1.

### Western blot analysis.

Western blot analysis used standard methods and is detailed in Text S1.

### Biofilm production in 96-well plate.

Biofilm production and imaging procedures followed previous published methods with minor modifications (33), as detailed in Text S1.

### β-Galactosidase activity assay.

To assay expression levels of LacZ, we used two different methods: an 5-bromo-4-chloro-3-indolyl-β-d-galactopyranoside (X-Gal) overlay plate assay and a β-galactosidase extract assay. For the X-Gal overlay assay, cells grown in YPD overnight at 30°C were spotted on a RPMI plus 10% FBS plate and incubated for 24 h at 37°C. Then, agarose containing X-Gal (Thermo Scientific catalog number R0941) and Z-buffer (60 mM Na_2_HPO_4_, 60 mM NaH_2_PO_4_, 10 mM KCl, and 1 mM MgSO_4_) was overlaid on the plate and incubated until colonies turned blue (34, 35). For the extract assay, cells were grown in 25 mL of RPMI with 10% FBS at 37°C for 24 h, washed with phosphate-buffered saline twice, and lysed with 0.25 M Tris (pH 8.0) using a bead beater. The X-Gal assay kit (Invitrogen catalog number 45-0449) was used according to the manufacturer’s instructions.

### Data analysis software.

Transcriptome and ChIP-seq data were visualized using Integrative Genomics Viewer (IGV) v2.11.0 (36). Venn diagrams were constructed using a Venn diagrams tool (http://bioinformatics.psb.ugent.be/webtools/Venn/). A heatmap for gene expression analysis was constructed using MultiExperiment Viewer. Regulatory network was constructed using Cytoscape software v.3.9.1 (37). Biofilm and filamentation images were processed using Image J (Fiji) (38). The PrLD prediction analysis was performed by using prion-like amino acid composition (http://plaac.wi.mit.edu) (39).

### Statistics.

Statistical analysis using the Benjamini-Hochberg procedure for data on the NanoString platform was performed using nSolver v4.0. Graph construction and statistical analysis were performed using GraphPad Prism version 9.2.0 (GraphPad Software, Inc., La Jolla).

### Data availability.

Processed RNA-seq and ChIP-seq data are available in the supplemental Data Set files; raw data are available through NCBI SRA with accession numbers PRJNA857655 (WT versus *efg1*Δ/Δ RNA-seq), PRJNA848228 (+*WOR1* versus +empty vector RNA-seq), and PRJNA849610 (Efg1-HA ChIP-seq).
