# Supplementary figures and images for: Collaboration between Antagonistic Cell Type Regulators Governs Natural Variation in the Candida albicans Biofilm and Hyphal Gene Expression Network
Source: mBio. 2022 Aug 22;13(5):e01937-22. doi: 10.1128/mbio.01937-22 (PMC9600859; doi:10.1128/mbio.01937-22)

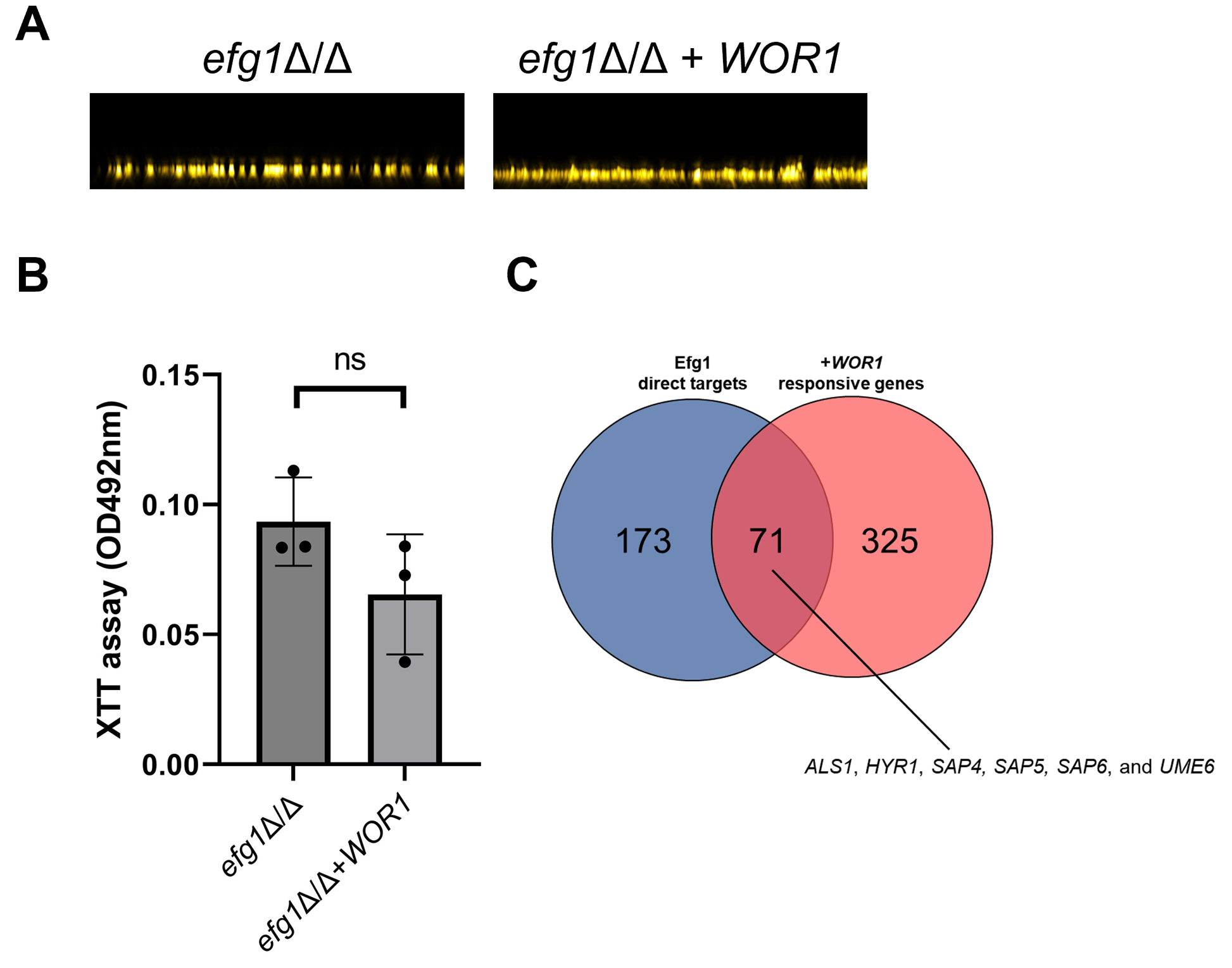

Supplement: FIG S1 [file mbio.01937-22-s0007.tif]

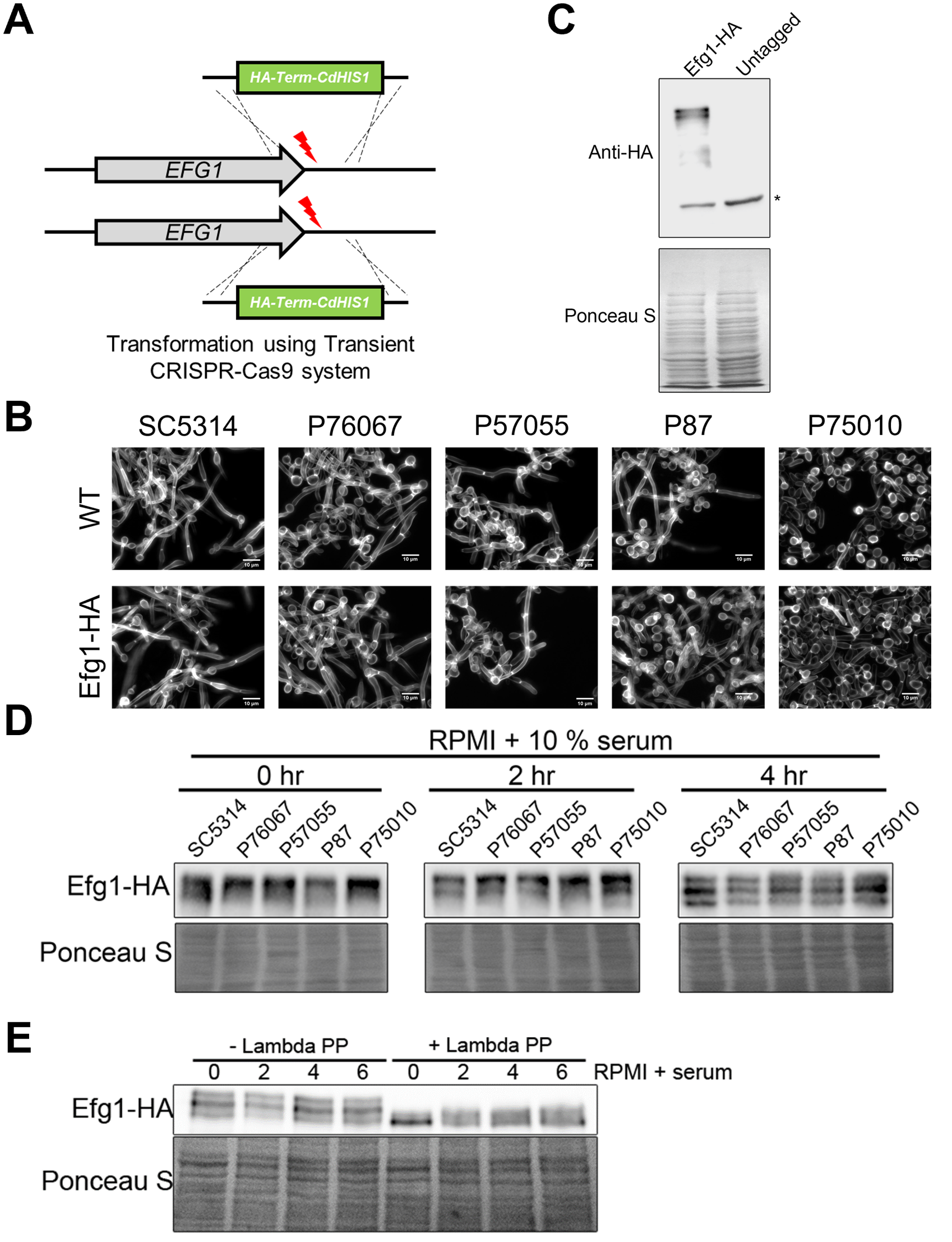

Supplement: FIG S2 [file mbio.01937-22-s0008.tif]
