# Supplementary material for: Collaboration between Antagonistic Cell Type Regulators Governs Natural Variation in the Candida albicans Biofilm and Hyphal Gene Expression Network
Source: mBio. 2022 Aug 22;13(5):e01937-22. doi: 10.1128/mbio.01937-22 (PMC9600859; doi:10.1128/mbio.01937-22)
Supplement: TEXT S1 [file mbio.01937-22-s0009.docx]

**Detailed Materials and Methods**

**Primers and plasmid construction**

To construct plasmid pED3-HA, the 3x HA with *ACT1* terminator sequences were amplified from SC5314 genomic DNA with primers ACT1_F_HA_HindIII and Act1_R_SacI. The amplified fragments were digested with restriction enzyme HindIII and SacI and inserted at the HindIII and SacI restriction sites in the plasmid pSN52 ^1^. The resulting plasmid was confirmed by using Sanger sequencing.

To construct plasmids for LacZ activity assay, *CHT2* promoter regions were amplified from SC5314 and P75010 genomic DNA with primers Reporter_Efg1-1_F and Reporter_Efg1-1_R (*P_CHT2_*-1), Reporter_Efg1-2_F and Reporter_Efg1-2_R (*P_CHT2_*-2), Reporter_Efg1-3_F and Reporter_Efg1-3_R (*P_CHT2_*-3), Reporter_Efg1-1_F and Reporter_Efg1-2_R (*P_CHT2_*-4), Reporter_Efg1-2_F and Reporter_Efg1-3_R (*P_CHT2_*-5), or Reporter_Efg1-1_F and Reporter_Efg1-3_R (*P_CHT2_*-6). The amplified fragments were digested with ApaI and inserted at the ApaI restriction site in the plasmid pCR-opLacZ-MDR1-HIS1 (unpublished data) ^2^. The resulting plasmids were confirmed by using Sanger sequencing.

To construct a plasmid pED20, *WOR1* terminator sequence from SC5314 genomic DNA was amplified with primers Wor1term_F_AatII and Wor1term_R_NcoI. The DNA fragments were digested with AatII and NcoI and inserted at the AatII and NcoI restriction sites in the plasmid pNAT ^3^. The resulting plasmid was confirmed by using Sanger sequencing.

All primer sequences and plasmids used in this study are listed in tables below.

**Strain construction**

To manipulate *C. albicans* genome, the transient CRISPR-Cas9 system was employed as previously described in detail ^3^. Generally, the Cas9 cassette was amplified from the plasmid pV1093, and each of sgRNA cassette was generated by using split-joint PCR with “sgRNA/F YFG1” and “SNR52/R YFG1” as previously described in detail ^3,4^.

To construct Efg1-HA tagged strains in 5 clinical isolates, the plasmid pED3-HA was used for amplification of 3x HA tag cassette containing *CdHIS1* marker with primers “EFG1_F-HA” and “Efg1_R-HA” (S2A Figure). To construct Efg1-HA in P75010 strain background, linker sequences (GGGSGGGS) was inserted between *EFG1* ORF and 3x HA tag sequence. The Efg1-linker-HA cassette was amplified with primers "Efg1_5'ext_F" and "Efg1_5'ext_R" for 5' flanking region from P75010 genomic DNA, "Efg1_linker_F" and "Efg1-HA_R" for 3x HA tag cassette from the plasmid pED3-HA and "Efg1_3'ext_F" and "Efg1_3'ext_R" for 3' flanking region from P75010 genomic DNA. Subsequent fragments were used for overlap extension PCR with primers "EFG1_TAG_F" and "EFG1_TAG_R". In *his1*Δ/Δ mutant of each isolate background, Efg1 downstream regions, 297 bp (SC5314 and P76067) or 303 bp (P57055, P87, and P75010), were replaced with the Efg1-HA cassette by using Cas9 and Efg1term sgRNA cassette. Transformation was conducted with approximately 3 μg of Cas9, 1 μg of sgRNA and 3 μg of Efg1-HA repair template. Transformants were screened on CSM lacking histidine plate, and candidates were genotyped by PCR using primers “Efg1_5'ext_F” and “ACT1term int/R” for presence of Efg1-tag cassette and using primers “Efg1_5'ext_F” and “Efg1_3'ext_R” for absence of targeted *EFG1* downstream region at the *EFG1* locus. To test whether the *EFG1-HA* alleles are functional, strains were assessed by hypha formation assays (S2B,C Figure). In addition, Efg1-HA protein abundance (S2D Figure) and phosphorylation state (S2E Figure) were tested among the clinical isolates under the conditions used for RNA-seq and ChIP-seq.

To construct *mdr1*Δ/Δ mutant in WT and *efg1*Δ/Δ mutant of P75010 strain background, the MDR1 deletion cassette was amplified from the plasmid pNAT with primers “Mdr1_deletion_NAT_F” and “Mdr1_deletion_NAT_R”. Each strain was transformed with approximately 3 μg of Cas9, 1 μg of MDR1-5 sgRNA and 3 μg of *NAT1* repair template. Transformants were screened on YPD containing 400 μg/mL nourseothricin, and candidates were genotyped by PCR using primers “MDR1 check up/F” and “MDR1 check int/R” for absence of *MDR1* ORF and using primers “MDR1 check up/F” and “NAT CRIME/R” for presence of *NAT1* marker at the *MDR1* locus.

To construct ectopic expression strains in both WT and *efg1*Δ/Δ mutant in the P75010 strain background, we replace the *MDR1* ORF region with each TF allele of P75010 strain using our concatemer assembly method ^5^.

The *ADR1^P75010^* allele containing 1951bp of *ADR1* upstream region, the *ADR1* ORF, and 533bp of *ADR1* downstream region was amplified from P75010 genomic DNA with primers “ADR1_Far upstream_F for MDR1_F” and “ADR1 3'-> pNAT 5'R” which are containing concatenating homology to selective marker gene, *NAT1*. The *NAT1* cassette was amplified from the plasmid pNAT with primers “pNAT for adap/F” and “pNAT 3'R -> MDR1 adap/R”. Transformation was conducted with approximately 3 μg of Cas9, 1 μg of MDR1-5 sgRNA, 3 μg of *ADR1* cassette, and 3 μg of *NAT1* cassette. Transformants were screened on YPD plate containing 400 μg/mL nourseothricin, and candidate colonies were genotyped by PCR using primers “MDR1 check up/F” and “MDR1 check int/R” for absence of the MDR1 ORF and using primers “MDR1 check up/F” and “ADR1 check int/R3” for presence of repair template at the *MDR1* locus.

The *BRG1^P75010^* allele containing 1637bp of *BRG1* upstream region, the *BRG1* ORF, and 348bp of *BRG1* downstream region was amplified from P75010 genomic DNA with primers “BRG1_Far upstream_F for MDR1_F” and “BRG1 3'-> pNAT 5'R” which are containing concatenating homology to selective marker gene, *NAT1*. The *NAT1* cassette was amplified from the plasmid pNAT with primers “pNAT for adap/F” and “pNAT 3'R -> MDR1 adap/R”. Transformation was conducted with approximately 3 μg of Cas9, 1 μg of MDR1-5 sgRNA, 3 μg of *BRG1* cassette, and 3 μg of *NAT1* cassette. Transformants were screened on YPD plate containing 400 μg/mL nourseothricin, and candidate colonies were genotyped by PCR using primers “MDR1 check up/F” and “MDR1 check int/R” for absence of the *MDR1* ORF and using primers “MDR1 check up/F” and “BRG1 check int/R” for presence of repair template at the *MDR1* locus.

The *TEC1^P75010^* allele containing 2071bp of *TEC1* upstream region, the *TEC1* ORF, and 854bp of *TEC1* downstream region was amplified from P75010 genomic DNA with primers “TEC1_Far upstream_F for MDR1_F” and “TEC1 3'-> pNAT 5'R” which are containing concatenating homology to selective marker gene, *NAT1*. The *NAT1* cassette was amplified from the plasmid pNAT with primers “pNAT for adap/F” and “pNAT 3'R -> MDR1 adap/R”. Transformation was conducted with approximately 3 μg of Cas9, 1 μg of MDR1-5 sgRNA, 3 μg of *TEC1* cassette, and 3 μg of *NAT1* cassette. Transformants were screened on YPD plate containing 400 μg/mL nourseothricin, and candidate colonies were genotyped by PCR using primers “MDR1 check up/F” and “MDR1 check int/R” for absence of the MDR1 ORF and using primers “MDR1 check up/F” and “TEC1 check int/R” for presence of repair template at the *MDR1* locus.

The *WOR1^P75010^* allele containing 2150bp of *WOR1* upstream region, the *WOR1* ORF, and 555bp of *WOR1* downstream region was amplified from P75010 genomic DNA with primers “WOR1_Far upstream_F for MDR1_F” and “WOR1 3'-> pNAT 5'R” which are containing concatenating homology to selective marker gene, *NAT1*. The *NAT1* cassette was amplified from the plasmid pNAT with primers “pNAT for adap/F” and “pNAT 3'R -> MDR1 adap/R”. Transformation was conducted with approximately 3 μg of Cas9, 1 μg of MDR1-5 sgRNA, 3 μg of *WOR1* cassette, and 3 μg of *NAT1* cassette. Transformants were screened on YPD plate containing 400 μg/mL nourseothricin, and candidate colonies were genotyped by PCR using primers “MDR1 check up/F” and “MDR1 check int/R” for absence of the MDR1 ORF and using primers “MDR1 check up/F” and “WOR1 check int/R” for presence of repair template at the *MDR1* locus.

The *ZFU2^P75010^* allele containing 1803bp of *ZFU2* upstream region, the *ZFU2* ORF, and 398bp of *ZFU2* downstream region was amplified from P75010 genomic DNA with primers “ZFU2_Far upstream_F for MDR1_F” and “ZFU2 3'-> pNAT 5'R” which are containing concatenating homology to selective marker gene, *NAT1*. The *NAT1* cassette was amplified from the plasmid pNAT with primers “pNAT for adap/F” and “pNAT 3'R -> MDR1 adap/R”. Transformation was conducted with approximately 3 μg of Cas9, 1 μg of MDR1-5 sgRNA, 3 μg of *ZFU2* cassette, and 3 μg of *NAT1* cassette. Transformants were screened on YPD plate containing 400 μg/mL nourseothricin, and candidate colonies were genotyped by PCR using primers “MDR1 check up/F” and “MDR1 check int/R” for absence of the MDR1 ORF and using primers “MDR1 check up/F” and “ZFU2 check int/R2” for presence of repair template at the *MDR1* locus.

The *WOR1^P75010^* allele containing 2150bp of *WOR1* upstream region and the *WOR1* DBD was amplified from P75010 genomic DNA with primers “WOR1_Far upstream_F for MDR1_F” and “Wor1_5prime_R_for PrLD” which are containing concatenating homology to Wor1 PrLD cassettes. The PrLD of Wor1 with YF-to-S or DE-to-A substitutions were synthesized from IDT, and the plasmid pED21 or pED22 contain Wor1 PrLD with YF-to-S or DE-to-A substitutions, respectively. To construct the Wor1 PrLD with amino acid substitutions, PrLD with YF-to-A substitutions were amplified from pED21 with primers “Wor1_PrLD_Start_F” and “DetoA_R” for 5′ region and “YFtoS_repair_F” and “YFtoS_R” for 3′ region of Wor1 PrLD. The PrLD with DE-to-A substitutions were amplified from pED21 with primers “Wor1_PrLD_Start_F” and “DetoA_R”. The cassette containing *WOR1* terminator sequence with *NAT1* marker was amplified using the plasmid pED20 with primers “Wor1term-NAT_F” and “pNAT 3'R -> MDR1 adap/R”. Transformation was conducted with approximately 3 μg of Cas9, 1 μg of MDR1-5 sgRNA, 3 μg of *WOR1* DBD cassette, 3 μg of *WOR1* PrLD substitutions cassette and 3 μg of *NAT1* cassette. Transformants were screened on YPD plate containing 400 μg/mL nourseothricin, and candidate colonies were genotyped by PCR using primers “MDR1 check up/F” and “MDR1 check int/R” for absence of the MDR1 ORF and using primers “MDR1 check up/F” and “WOR1 check int/R” for presence of repair template at the *MDR1* locus.

The *WOR1, BRG1, TEC1* and *EFG1* alleles of P75010 and SC5314 specify products that are at least are 98% identical, as illustrated in this BLASTP summary:

| **1. Wor1** | **Identities** | **Positives** | **Gaps** |
| --- | --- | --- | --- |
| SC5314 A allele vs P75010: | 782/786(99%) | 783/786(99%) | 0/786(0%) |
| SC5314 B allele vs P75010: | 780/786(99%) | 782/786(99%) | 0/786(0%) |
|  |  |  |  |
| **2. Brg1** | **Identities** | **Positives** | **Gaps** |
| SC5314 A allele vs P75010: | 410/418(98%) | 414/418(99%) | 0/418(0%) |
| SC5314 B allele vs P75010: | 417/419(99%) | 417/419(99%) | 1/419(0%) |
|  |  |  |  |
| **3. Tec1** | **Identities** | **Positives** | **Gaps** |
| SC5314 A allele vs P75010: | 736/744(99%) | 737/744(99%) | 3/744(0%) |
| SC5314 B allele vs P75010: | 736/744(99%) | 737/744(99%) | 3/744(0%) |
|  |  |  |  |
| **4. Efg1** | **Identities** | **Positives** | **Gaps** |
| SC5314 A allele vs P75010: | 550/557(99%) | 550/557(98%) | 6/557(1%) |
| SC5314 B allele vs P75010: | 553/557(99%) | 553/557(99%) | 2/557(0%) |

To construct LacZ reporter expression strains in both SC5314 and P75010 strain background, *CdHIS1* marker at the *EFG1* locus in *efg1*Δ/Δ mutants was deleted to construct histidine auxotrophic strains. A *NAT1* repair template was amplified from the plasmid pNAT with primers “Efg1-NAT_F” and “Efg1-NAT_R”. Each strain was transformed with approximately 3 μg of Cas9, 1 μg of r1 sgRNA and 3 μg of *NAT1* repair template. Transformants were screened on YPD containing 400 μg/mL nourseothricin, and candidates were genotyped by PCR using primers “Efg1_Check/F” and “HIS1 check int/R” for absence of *CdHIS1* marker, and primers “Efg1_Check/F” and “NAT CRIME/R” for presence of *NAT1* marker at the *EFG1* locus. Then, the plasmids containing fragmented *CHT2* promoter region were linearized by BstAPI and integrated at the *MDR1* locus by using the transient CRISPR-Cas9 system. Each strain was transformed with approximately 3 μg of Cas9, 1 μg of MDR1-5 sgRNA and 10 μg of linearized plasmid. Transformants were screened on CSM lacking histidine plate, and candidates were genotyped by PCR using primers “MDR1 check up/F” and “MDR1 check int/R” for absence of the *MDR1* ORF and using primers “HIS CRIME/F” and “MDR1 check down/R” for presence of repair template at the *MDR1* locus.

**Chromatin immunoprecipitation**

To extract soluble chromatin from *C. albicans*, stains grown in 5 mL YPD at 30°C for overnight were inoculated into 100 mL of RPMI + 10% serum to an OD_600_ of 0.2. Cells were then cultured at 37°C for 4 hours in a shaking incubator with 225 rpm, fixed with formaldehyde (1% final concentration) for 15 min at RT, and quenched with glycine (300 mM final concentration) for 10 min at RT. Cells were washed with ice-cold PBS twice and lysed with FA lysis buffer (50 mM HEPES-KOH, 140 mM NaCl, 1 mM EDTA, 1% Triton X-100, 0.1% Sodium deoxycholate, 0.1% SDS, 1 mM PMSF, and 1x proteinase inhibitor cocktail) and glass beads using bead-beater. Cell lysates were clarified by centrifugation and sonicated using Bioruptor sonicator (Diagenode) for 15 cycles. Then the lysates were clarified by centrifugation and used for immunoprecipitation as previously described method with minor modifications ^6^. Briefly, Dynabeads protein G (Invitrogen, 10003D) was conjugated with anti-HA antibody (Abcam, ab9110) and incubated with sheared chromatin samples at 4C for overnight with rotation. Beads were washed three times with FA lysis buffer and three times with FA lysis buffer containing 500 mM of NaCl using magnet strand (Invitrogen). Finally, beads were washed with TE (10 mM Tris-HCl, pH 8.0, and 1 mM EDTA) and resuspended with elution buffer (50 mM Tris-HCl, pH 8.0, 10 mM EDTA, and 1% SDS). Beads were then incubated at 65°C for 15 min, and eluted samples were incubated at 65°C for overnight. Each sample was treated with proteinase K (Invitrogen) and RNaseA (Invitrogen) at 50°C for 1.5 hr, and reverse-cross-linked DNA samples were purified using ethanol precipitation method with glycogen (Invitrogen). DNA quantification was performed by using Qubit dsDNA HS Assay Kit (Molecular probes).

**ChIP-seq library preparation**

ChIP-seq libraries were prepared in triplicate from each strain using the NEBNext® Ultra™ II DNA Library Prep Kit for Illumina® (New England Biolabs) according to the manufacturer’s instructions with the following modifications. For input DNA samples, 75 ng of DNA was used as starting template for the libraries. The adapters were diluted 1:10 and 7 PCR amplification cycles were performed. For IP DNA samples, the libraries were prepared with 1.6 ng of DNA as starting template, the adapters were diluted 1:25, and 12 PCR cycles were performed to amplify the library. The IP libraries were size selected by adding 36 μl of nuclease free water to 14 μl of library. Agencourt® AMPure® XP beads (Beckman Coulter) were added to a final volume of 75 μl and after magnetization the supernatant was transferred to a new tube containing 42.5 μl of beads. After magnetization, the pellet was twice washed with 80% EtOH and the library was eluted in 15 μl of nuclease free water. The libraries were pooled and sequenced (2 x 51 nt, paired-end) on a HiSeq 2000 Sequencing System (lllumina).

**Western blot analysis**

To extract total soluble protein, cells were grown in 25 mL of RPMI + 10% serum for 4 hours at 37°C and then lysed with FA lysis buffer (50 mM HEPES-KOH, 140 mM NaCl, 1 mM EDTA, 1% Triton X-100, 0.1% Sodium deoxycholate, 1 mM PMSF, and 1x proteinase inhibitor cocktail) with glass beads (Sigma) using bead-beater ^6^. The cell lysates were clarified using centrifugation at 14,000 rpm for 5min at 4°C, and total protein concentrations were measured by using Bradford assay (Bio-Rad). Total 40 μg of cell lysate from each strain was loaded and separated in 8% SDS polyacrylamide gel and transferred to nitrocellulose membrane (Bio-Rad). Ponceau S (Sigma) straining was used for confirmation of equal protein loading in each well. Membrane was blocked with 5% non-fat dry milk (Bio-Rad) in TBST (tris-buffered saline containing 0.05% Tween 20, pH7.4) for overnight at 4°C and washed with TBST. Then, the membrane was incubated with 1:5000 diluted anti-HA monoclonal mouse antibody (Roche; #11 583 816 001) in TBST for 1 hours at RT, washed with TBST, and incubated with 1:10000 diluted anti-mouse IgG-HRP (Santa Cruz Biotechnology; sc-516102) for 1 hour at RT followed by washing with TBST. Signals were developed by enhanced chemiluminescence (Thermo Fisher Scientific, #34580) and imaged using ChemStudio (analytik-jena, Thuringia, Germany).

**Biofilm production in 96-well plate**

Biofilm production and imaging were followed previous published methods with minor modifications ^7^. To assay biofilm formation in 96-well plate, cells were grown in 5 mL of YPD for overnight at 30°C, then cells were transferred to 100 μL of pre-warmed RPMI with 10% FBS to achieve an OD_600_ of 0.5 in 96-well plate (Greiner, 655090). The cells were incubated in a shaker incubator at 37°C for 90min with mild-shaking (60 rpm) to allow for adherence to bottom of 96-well, then each well was gently washed twice with PBS to remove non-adhered cells. One-hundred microliter of pre-warmed RPMI with 10% FBS was added into each well, and cells were allowed to form biofilm in a shaker incubator with 60 rpm at 37°C for 24 hours. In next day, medium was carefully discarded from each well, biofilms were fixed by incubation with 100 μL of 4% formaldehyde in PBS solution for 1 hour and then gently washed twice with PBS. Subsequent fixed biofilms were stained with Calcofluor-white (200 μg/ml in PBS) for overnight at RT with mild shaking (60 rpm), then each well was gently washed twice with PBS. For clarification and refractive index matching, biofilms were incubated with 50% of 2,2′-Thiodiethanol (TDE) in PBS for 1 hour at RT and then 100% TDE was added to each biofilm. Biofilm were imaged by using a Confocal microscopy (Zeiss LSM710) with a Zeiss 40X/1.3 NA oil immersion objective.

**β-galactosidase activity assay**

To assay expression level of LacZ, we used two different methods which are X-gal overlay and β-galactosidase assay. For the X-gal overlay assay, cells grown in YPD for overnight at 30°C were spotted on RPMI + 10% FBS plate and incubated for 24 hours at 37°C. Then, agarose containing X-gal (Thermo Scientific, R0941) and Z-buffer (60 mM Na_2_HPO_4_, 60 mM NaH_2_PO_4_, 10 mM KCl, and 1 mM MgSO_4_) was overlayed on the plate and incubated until colonies turning blue ^8,9^. Second, cells were grown in 25 mL of RPMI + 10% FBS at 37°C for 24 hours, washed with PBS twice and lysed with 0.25 M Tris, pH 8.0 using bead beater. The X-gal assay kit (Invitrogen, 45-0449) was used according to the manufacturer’s instructions.

**Data analysis software**

Transcriptome and ChIP-seq data were visualized using Integrative genomics viewer (IGV) v2.11.0 ^10^. Venn diagrams were constructed using Venn Diagrams tool (http://bioinformatics.psb.ugent.be/webtools/Venn/). Heat-map for gene expression analysis was constructed using MultiExperiment Viewer (MeV). Regulatory network was constructed using Cytoscape software v.3.9.1 ^11^. Biofilm and filamentation images were processed using Image J (Fiji) ^12^. The PrLD prediction analysis was performed by using Prion-like amino acid composition (PLACC; http://plaac.wi.mit.edu) ^13^.

**Strains used in this study**

| **Strain** | **Parent Strain** | **Description / Genotype** | **Notes** |
| --- | --- | --- | --- |
| SC5314 | n/a | Candida albicans clinical isolates | WT |
| P76067 | n/a | Candida albicans clinical isolates | WT |
| P57055 | n/a | Candida albicans clinical isolates | WT |
| P87 | n/a | Candida albicans clinical isolates | WT |
| P75010 | n/a | Candida albicans clinical isolates | WT |
| SC5314 his1Δ/Δ | SC5314 | *his1Δ::r3NAT1r3/his1Δ::r3NAT1r3* | homozygous (Huang et al., 2019) |
| P76067 his1Δ/Δ | P76067 | *his1Δ::r3NAT1r3/his1Δ::r3NAT1r3* | homozygous (Huang et al., 2019) |
| P57055 his1Δ/Δ | P57055 | *his1Δ::r3NAT1r3/his1Δ::r3NAT1r3* | homozygous (Huang et al., 2019) |
| P87 his1Δ/Δ | P87 | *his1Δ::r3NAT1r3/his1Δ::r3NAT1r3* | homozygous (Huang et al., 2019) |
| P75010 his1Δ/Δ | P75010 | *his1Δ::r3NAT1r3/his1Δ::r3NAT1r3* | homozygous (Huang et al., 2019) |
| SC5314 efg1 Δ/Δ | SC5314 his1Δ/Δ | *efg1Δ::rHIS1r/efg1Δ::rHIS1r his1Δ::r3/his1Δ::r3* | homozygous (Huang et al., 2019) |
| P76067 efg1 Δ/Δ | P76067 his1Δ/Δ | *efg1Δ::rHIS1r/efg1Δ::rHIS1r his1Δ::r3/his1Δ::r3* | homozygous (Huang et al., 2019) |
| P57055 efg1 Δ/Δ | P57055 his1Δ/Δ | *efg1Δ::rHIS1r/efg1Δ::rHIS1r his1Δ::r3/his1Δ::r3* | homozygous (Huang et al., 2019) |
| P87 efg1 Δ/Δ | P87 his1Δ/Δ | *efg1Δ::rHIS1r/efg1Δ::rHIS1r his1Δ::r3/his1Δ::r3* | homozygous (Huang et al., 2019) |
| P75010 efg1 Δ/Δ | P75010 his1Δ/Δ | *efg1Δ::rHIS1r/efg1Δ::rHIS1r his1Δ::r3/his1Δ::r3* | homozygous (Huang et al., 2019) |
| EFG1-HA-ACT1 term | SC5314 his1Δ/Δ | *EFG1-3XHA-ACT1term-HIS1/EFG1-3XHA-ACT1term-HIS1, his1Δ::r3NAT1r3/his1Δ::r3NAT1r3* | homozygous |
| EFG1-HA-ACT1 term | P76067 his1Δ/Δ | *EFG1-3XHA-ACT1term-HIS1/EFG1-3XHA-ACT1term-HIS1, his1Δ::r3NAT1r3/his1Δ::r3NAT1r3* | homozygous |
| EFG1-linker-HA | P75010 his1Δ/Δ | *EFG1-linker-3XHA-ACT1term-HIS1/EFG1-3XHA-ACT1term-HIS1, his1Δ::r3NAT1r3/his1Δ::r3NAT1r3* | homozygous |
| EFG1-HA-ACT1 term | P57055 his1Δ/Δ | *EFG1-3XHA-ACT1term-HIS1/EFG1-3XHA-ACT1term-HIS1, his1Δ::r3NAT1r3/his1Δ::r3NAT1r3* | homozygous |
| EFG1-HA-ACT1 term | P87 his1Δ/Δ | *EFG1-3XHA-ACT1term-HIS1/EFG1-3XHA-ACT1term-HIS1, his1Δ::r3NAT1r3/his1Δ::r3NAT1r3 (extended homology method using overlap PCR)* | homozygous |
| mdr1::BRG | P75010 | *mdr1Δ::BRG1-NAT1/mdr1Δ::BRG1-NAT1 (extended homology method)* | homozygous |
| mdr1::BRG1, efg1Δ/Δ | P75010 efg1Δ/Δ | *mdr1Δ::BRG1-NAT/mdr1Δ::BRG1-NAT, efg1Δ::rHIS1r/efg1Δ::rHIS1r his1Δ::r3/his1Δ::r3* | homozygous |
| mdr1::WOR1 | P75010 WT | *mdr1Δ::WOR1-NAT/mdr1Δ::WOR1-NAT* | homozygous |
| mdr1:WOR1-P75010, efg1Δ/Δ | P75010 efg1Δ/Δ | *mdr1Δ::WOR1-NAT/mdr1Δ::WOR1-NAT, efg1Δ::rHIS1r/Δ efg1Δ::rHIS1r his1Δ::r3/his1Δ::r3* | homozygous |
| mdr1:ZFU2-P75010 | P75010 WT | *mdr1Δ::ZFU2-NAT/mdr1Δ::ZFU2-NAT* | homozygous |
| mdr1:ZFU2-P75010, efg1Δ/Δ | P75010 efg1Δ/Δ | *mdr1Δ::ZFU2-NAT/mdr1Δ::ZFU2-NAT, efg1Δ::rHIS1r/Δ efg1Δ::rHIS1r his1Δ::r3/his1Δ::r3* | homozygous |
| mdr1::ADR1 | P75010 WT | *mdr1Δ::ADR1-NAT/mdr1Δ::ADR1-NAT* | homozygous |
| mdr1::ADR1, efg1Δ/Δ | P75010 efg1Δ/Δ | *mdr1Δ::ADR1-NAT/mdr1Δ::ADR1-NAT, efg1Δ::rHIS1r/Δ efg1Δ::rHIS1r his1Δ::r3/his1Δ::r3* | homozygous |
| mdr1:TEC1 | P75010 WT | *mdr1Δ::TEC1--NAT/mdr1Δ::TEC1--NAT* | homozygous |
| mdr1:TEC1, efg1Δ/Δ | P75010 efg1Δ/Δ | *mdr1Δ::TEC1-NAT/mdr1Δ::TEC1-NAT, efg1Δ::rHIS1r/Δ efg1Δ::rHIS1r his1Δ::r3/his1Δ::r3* | homozygous |
| mdr1Δ/Δ | P75010 WT | *mdr1Δ::NAT/mdr1Δ::NAT* | homozygous |
| mdr1Δ/Δ, efg1Δ/Δ | P75010 efg1Δ/Δ | *mdr1Δ::NAT/mdr1Δ::NAT, efg1Δ::rHIS1r/efg1Δ::rHIS1r his1Δ::r3/his1Δ::r3* | homozygous |
| WT + Wor1 (YF to S) | P75010 WT | *mdr1Δ::WOR1(YF to S)-NAT/mdr1Δ::WOR1(YF to S)-NAT* | homozygous |
| efg1Δ/Δ + Wor1 (YF to S) | P75010 efg1Δ/Δ | *mdr1Δ::WOR1(YF to S)-NAT/mdr1Δ::WOR1(YF to S)-NAT, efg1Δ::rHIS1r/Δ efg1Δ::rHIS1r his1Δ::r3/his1Δ::r3* | homozygous |
| WT + Wor1 (DE to A) | P75010 WT | *mdr1Δ::WOR1(DE to A)-NAT/mdr1Δ::WOR1(DE to A)-NAT* | homozygous |
| efg1Δ/Δ + Wor1 (DE to A) | P75010 efg1Δ/Δ | *mdr1Δ::WOR1(DE to A)-NAT/mdr1Δ::WOR1(DE to A)-NAT, efg1Δ::rHIS1r/Δ efg1Δ::rHIS1r his1Δ::r3/his1Δ::r3* | homozygous |

**Primers used in this study**

| **Primer Name:** | **Sequence:** |
| --- | --- |
| **General primers** |  |
| NAT1 CRIME/R | CACCATGACCTCTATGTTCTGG |
| NAT1 CRIME/F | CAGACGCGTTGAATTGTCC |
| HIS1 CRIME/F | gcgcaagaagcctcaact |
| HIS1 CRIME/R | gagctacagggcttgacc |
| CdHIS1 Check Int/R | ggctgatttgtctttacatcg |
| **HA Cloning** |  |
| ACT1_F_FLAG_HindIII | GGATCTAAGCTTGACTACAAAGACCATGACGGTGATTATAAAGATCATGACATCGACTACAAGGATGACGATGACAAGTAAGAGTGAAATTCTGGAAATCTGG |
| ACT1_F_HA_HindIII | GGATCTAAGCTTtacccatacgatgttcctgactatgcgggctatccctatgacgtcccggactatgcaggatcctatccatatgacgttccagattacgctTAAGAGTGAAATTCTGGAAATCTGG |
| Act1_R_SacI | AGATCCGAGCTCGATGTTGCTAGATTATGGTCGAC |
| **Efg1-Tag** |  |
| EFG1_F-FLAG | TGACTCAAGGTTCAGTTCACCCTTCACCCCAACAACATCAAGCTAATCAATCAGCTAGCACTGTTGCCAAAGAAGAAAAGGACTACAAAGACCATGACGGTG |
| EFG1_F-HA | TGACTCAAGGTTCAGTTCACCCTTCACCCCAACAACATCAAGCTAATCAATCAGCTAGCACTGTTGCCAAAGAAGAAAAGTACCCATACGATGTTCCTGACTATG |
| SNR52/R_EFG1term | TCTGCTTTCTGCCATAAATTCAAATTAAAAATAGTTTACGCAAGTC |
| sgRNA/F_EFG1term | AATTTATGGCAGAAAGCAGAGTTTTAGAGCTAGAAATAGCAAGTTAAA |
| EFG1-tag_confirm_F | CACCTGCATCACAACCAGGTTCTA |
| EFG1-tag_confirm_R | CGTTCATGTCAATGGATTTGGGAG |
| EFG1_3UTR_R | GCTCGTGCTTGGTACTTGATGTC |
| SNR52/R_EFG1_2 | AATAGTATAAATTCGTTCAT CAAATTAAAAATAGTTTACGCAAGTC |
| sgRNA/F_EFG1_2 | ATGAACGAATTTATACTATT GTTTTAGAGCTAGAAATAGCAAGTTAAA |
| Efg1_R-HA | AACGTCGTGACTGGGAAACTATGTATATGTATATATATGTGTGTAAGTCATTGTCTTTTCCATTTTCTTTTCCATTTTCTTTTCTTTTTAGTTTTGTT |
| Efg1_Tag_F | CACCTGCATCACAACCAGGTTCTA |
| Efg1-tag_confirm_R | CGTTCATGTCAATGGATTTGGGAG |
| Act1term int/R | ACAAAACCAGATTTCCAGATTTCCAGAA |
| Efg1_linker_F | CACCCCAACAACATCAAGCTAATCAATCAGCTAGCACTGTTGCCAAAGAAGAAAAG GGAGGTGGAGGTTCTGGTGGAGGTGGTTCA TACCCATACGATGTTCCTGACTATG |
| Efg1_5'ext_F | CCAACAGCAGTATCCTCAACAGTATG |
| Efg1_5'ext_R | CTTTTCTTCTTTGGCAACAGTGCTAG |
| Efg1_3'ext_F | GTGTGTAAGTCATTGTCTTTTCCATTTTC |
| Efg1_3'ext_R | ACTTAGAGTTGACTCTACCACAGTTAGC |
| Efg1_Tag_R | GAGATGGCATACTTACCACAATGCAC |
| **MDR1 deletion** |  |
| MDR1 check up/F | TGGGTGTTGCTACCAGTTAATCACAACG |
| MDR1 check int/R | AGCACCCAAACTCCAAGCG |
| MDR1 check int/F | ATGGTGATGATGATCCCGAAAACC |
| Mdr1_deletion_NAT_F | CTTTTTTTTATTCCGTAACAATCATATTATAATTTTACATTGCCCCAATAGCAATACATATACTTACATAGAACTTCATA GACATGGAGGCCCAGAATACCCT |
| Mdr1_deletion_NAT_R | AGAATCAGTCCTTTTCTCTTTTTAATTATTGATTAATGTATCTATAACACGATATATCTATAGGAAAACAATGACACCTC TGATTGCGTTAGTATCGAATCGACAG |
| MDR1 check up/F2 | GCTCGTTTAGTTGTTCCCAATCGC |
| MDR1 check down/R | TAAATAAACACAGGCTAATGAGAACATTGTGT |
| sgRNA/F MDR1-5 | tgtggtacccaattcaacga GTTTTAGAGCTAGAAATAGCAAGTTAAA |
| SNR52/R MDR1-5 | tcgttgaattgggtaccaca CAAATTAAAAATAGTTTACGCAAGTC |
| **BRG1 ectopic** |  |
| BRG1 Check Up/F | TGCAGCTTTTGTACTACATTTGG |
| BRG1 712 3’R->pNAT 5’/R | CGACGTCGGGCCCAATTCGCCCTATAGTGAGTCGTATTACAATTCACTGGCCGTCGTTTTACAACGTCGTGACTGGGAA ATTATGAATCGGTTGCAAATCGC |
| pNAT for adap/F | TTTCCCAGTCACGACGTT |
| pNAT 3'R -> MDR1 adap/R | AGAATCAGTCCTTTTCTCTTTTTAATTATTGATTAATGTATCTATAACACGATATATCTATAGGAAAACAATGACACCTC GTGGAATTGTGAGCGGATA |
| BRG1 Check Int/R | GGTATTGATTACTCTTGTCACTGG |
| BRG1 FarUp/F | ttacggcatcacaggcg |
| BRG1 FarDown/R | ttgctatcaccaacatttctgc |
| BRG1 check int/R | GGTATTGATTACTCTTGTCACTGG |
| BRG1 1641 5’F->MDR1 up/F | CTTTTTTTTATTCCGTAACAATCATATTATAATTTTACATTGCCCCAATAGCAATACATATACTTACATAGAACTTCATA TTTAAGGAACGGATATTTACCAGTCG |
| BRG1 712 3’R->pNAT 5’/R | CGACGTCGGGCCCAATTCGCCCTATAGTGAGTCGTATTACAATTCACTGGCCGTCGTTTTACAACGTCGTGACTGGGAAA TATTGTTTTAGTTGCCGCCTCAT |
| **TEC1 ectopic** |  |
| TEC1 3'-> pNAT 5'R | CGACGTCGGGCCCAATTCGCCCTATAGTGAGTCGTATTACAATTCACTGGCCGTCGTTTTACAACGTCGTGACTGGGAAA TGGACCCTTATAGGCATTGATGCA |
| TEC1_Farupstream_F for MDR1_F | CTTTTTTTTATTCCTTAACAATCATATTATAATTTTATATTGCCCCAATAGCAATACATATACTTACATAGAACTTCATA ACCCAATATCCAGTTTCACCTAAAACC |
| Tec1 check/up | ATACCTCACACCAACTCACACG |
| Tec1 check int/R | GACCAGATGTCAGTGTCTTGGA |
| **WOR1 ectopic** |  |
| WOR1 3'-> pNAT 5'R | CGACGTCGGGCCCAATTCGCCCTATAGTGAGTCGTATTACAATTCACTGGCCGTCGTTTTACAACGTCGTGACTGGGAAA GCCAATTCGTTCAGATATTCATACATCCA |
| WOR1_Farupstream_F for MDR1_F | CTTTTTTTTATTCCTTAACAATCATATTATAATTTTATATTGCCCCAATAGCAATACATATACTTACATAGAACTTCATA TCTGTCACATTCGAATGTCAGATAGAC |
| Wor1_Far_UP | TCCAAAACCCTGCCTTTCCTGTATT |
| Wor1_Far_Down | ATGGTCAAGGCGTCATCATATCATTCA |
| WOR1_3'_check_R | GGTATGATGATTTTCTGGATTTCCGTGT |
| Wor1_int/F | TGGTGGTCAAGGATATGCTACAGATGC |
| Wor1_3'_far_checkR | TAGTTTTGTTTATCTTGGAGTAATCGGAGT |
| Wor1_3'_far_checkR2 | AGCTCTATGAGAATTGATGGTTGTTAGACT |
| Wor1 check int/R | CCAGTATAATCTGGTTCATTGACATTGT |
| **ADR1 ectopic** |  |
| ADR1 3'-> pNAT 5'R | CGACGTCGGGCCCAATTCGCCCTATAGTGAGTCGTATTACAATTCACTGGCCGTCGTTTTACAACGTCGTGACTGGGAAA ATGCCAACAATTATCACAACAACCA |
| ADR1_Farupstream_F for MDR1_F | CTTTTTTTTATTCCTTAACAATCATATTATAATTTTATATTGCCCCAATAGCAATACATATACTTACATAGAACTTCATA TTATTTCCCACACGCGGTAATGACC |
| Adr1 check int/R3 | TCATGTTCAATTTCTTGTGTGATAGGCGA |
| Adr1_int/F | ACCAAATCAGTCTCTGCTCGGGTTATC |
| Adr1_Far_UP | TGCGACTTTTGAATTGTGAACGTTCTC |
| Adr1_Far_Down | CCACTTGACGAACCATCTGCCAGAAT |
| Adr1_upstream_R | ACCAAATCTACATTGGCCAATCAACATCA |
| ADR1 3'-> pNAT 5'R | CGACGTCGGGCCCAATTCGCCCTATAGTGAGTCGTATTACAATTCACTGGCCGTCGTTTTACAACGTCGTGACTGGGAAA AGCAGCAATGTACCACAGTATGATGGCA |
| Adr1_int/F2 | GCAGATCATGTAACACCTCCAGTAAACG |
| **ZFU2 ectopic** |  |
| Zfu2 3’R->pNAT 5’/R | CGACGTCGGGCCCAATTCGCCCTATAGTGAGTCGTATTACAATTCACTGGCCGTCGTTTTACAACGTCGTGACTGGGAAA TATAGGTTGATGAACACACAAAACACAA |
| pNAT-Zfu2down | TACTTCTACATCTTCTCCAACTATTAAGCCATGATATTGAAAGATATAGACTTAAGGCAATGTGTATGTGTGTATGTGTG GTGGAATTGTGAGCGGATA |
| Zfu2_checkup | GCTTTAACCCGCCTTAGCTGGT |
| Zfu2 check int/R | AATCGGAAGATGATGTGAAAGTATCA |
| ZFU2 3'-> pNAT 5'R | CGACGTCGGGCCCAATTCGCCCTATAGTGAGTCGTATTACAATTCACTGGCCGTCGTTTTACAACGTCGTGACTGGGAAA TATAGGTTGATGAACACACAAAACACAA |
| ZFU2_Farupstream_F for MDR1_F | CTTTTTTTTATTCCTTAACAATCATATTATAATTTTATATTGCCCCAATAGCAATACATATACTTACATAGAACTTCATA TCCTTCGGGGTCTCTTAAAGAAGTG |
| Zfu2 check int/R2 | CGGCAGAGAATATTCTGTCATTATTGTGGTG |
| Zfu2_int/F | AGAAGAACGGTGAAACGATCAAGAAATGG |
| ZFU2_Farupstream_F for MDR1_F2 | CTTTTTTTTATTCCTTAACAATCATATTATAATTTTATATTGCCCCAATAGCAATACATATACTTACATAGAACTTCATA ACCCTAAATTTTCCACGGCTTGATTTAAAT |
| **Sequencing primers** |  |
| Efg1 seq check/F | CCAACCCTTAACCCATTAACGAATTAAGA |
| Efg1 seq check/F2 | GCAGCAGCAGCAACAATATGATTACA |
| Efg1 seq check/R | TTGAGGATACTGCTGTTGGTTGTAAGT |
| **PrLD mutation** |  |
| Wor1term_F_AatII | GGATCT GACGTC TTGAATTAATACAGTGATTCAGTTATTATCTTGGGG |
| Wor1term_R_NcoI | GGATCT CCATGG TGGATGTATGAATATCTGAACGAATTGGC |
| Wor1dC_DBD_R | CTAACTTCCTGCAACTGAAGAAGAATGATGA |
| Wor1dC-WOR1_term_F | ATATTCAACAACAACAACAACAACACCAACTTCAACACCAACCACTTCTTCATCATTCTTCTTCAGTTGCAGGAAGTTAG TTGAATTAATACAGTGATTCAGTTATTATCTTGGGG |
| Wor1_PrLD_F | GACAAGAAGAAGAAAAAGAAGCGTAAATTTGGTCCCGACGACGAGTACGACCACAATGTCAATGAACCAGATTATACTGG TGGGTATGGTAACCACCTTCATAAT |
| DetoA_R | CATATATAAGATATACCAAATGTAAAAAAAAACACCTGAATAAGCCCCAAGATAATAACTGAATCACTGTATTAATTCAA CTAAGTACCGGTGTAATACGACCCAG |
| YFtoS_R | CATATATAAGATATACCAAATGTAAAAAAAAACACCTGAATAAGCCCCAAGATAATAACTGAATCACTGTATTAATTCAA CTAAGTACCGGTGGAAGACGACCCAG |
| WOR1_DBDint/F | TCATTAGGAGGCAAAGCTCCCATT |
| Wor1_term_int/R | TGCGGCAATACTACAATTCCCTTCATGA |
| Wor1_PrLD_int/R | AGTAGAGGTTGCACCGGAAATGGTA |
| Wor1term-NAT_F | TTGAATTAATACAGTGATTCAGTTATTATCTTGGGG |
| YFtoS_repair_R | AGAGCCTCCACTTGTAGTGCTTGTGGTGTTGGTACTGACTGCTGGTGGTCGATTGGTTGATGTAGAACTATTTGAATTGGAG CCAGAGTTATTTCCATTGCCATTACCAC |
| YFtoS_repair_F | CCAAACGTAAACCAAGTATTGTTAGTAATAGTGCCAGTGGAAGTGTTAGTGGTGGTAATGGCAATGGAAATAACTCTGGC TCCAATTCAAATAGTTCTACATCAACCAAT |
| YFtoS_repair_check/F | TGCCGCTAATCATCAGTCCCATTCAA |
| Wor1_5prime_R_for PrLD | ACTTCCTGCAACTGAAGAAGAATGATGA |
| Wor1_PrLD_Start_F | CCTATATTCAACAACAACAACAACAACACCAACTTCAACACCAACCACTTCTTCATCATTCTTCTTCAGTTGCAGGAAGT ACAACCAGTATTGTTAACAATAGTTTATCA |
| Wor1term_R_NcoI | GGATCT CCATGG GCCAATTCGTTCAGATATTCATACATCCA |
| **LacZ activity** |  |
| Reporter_Efg1-3_F | AGATCT GGGCCC TGGAAGTGATAGCAATGCCTACTAATGCT |
| Reporter_Efg1-3_R | AGATCT GGGCCC TCACAGTCAGTGGTCAATCTGTTTAGTT |
| Reporter_Efg1-2_F | AGATCT GGGCCC AACTAAACAGATTGACCACTGACTGTGA |
| Reporter_Efg1-2_R | AGATCT GGGCCC GACAAAGAGTTGTCTATGCATTCATCGT |
| Reporter_Efg1-1_F | AGATCT GGGCCC ACGATGAATGCATAGACAACTCTTTGTC |
| Reporter_Efg1-1_R | AGATCT GGGCCC TAACGAATGACTTGGGGTTAGTTGGA |
| LacZ_check/R | aatacttatgatctgagtgagcgtcaaca |
| opLacZ_Check/F | ATAGATAGCTCAATGGCTCATCTAAGCG |
| Efg1-NAT_F | AAAAAACAACCAACCAACCCTTAACCCATTAACGAATTAAGATTTGTTCTATTTGACTACCAAGAATATAACCCATATTA tttcccagtcacgacgtt |
| Efg1-NAT_R | TTTGTTAATGAAATATATGCTATAATCTAATTTGGAATTTATGGCAGAAAGCAGAAGGTGATGTACACAAATGATATTTA TGTGGAATTGTGAGCGGATA |
| Efg1_Check/F | AACTTGGTCCAAGAATTCATTACCAGGC |
| Efg1 far up/F | TCCAGCCAACCCACTTAACTTACAAT |
| HIS1 check int/R | ggctgatttgtctttacatcgtatcctcc |
| sgRNA/F r1 | GGGATCCACTAGTTCTAGAGGTTTTAGAGCTAGAAATAGCAAGTTAAA |
| SNR52/R r1 | CTCTAGAACTAGTGGATCCCCAAATTAAAAATAGTTTACGCAAGTC |

**Plasmids used in this study.**

| **Plasmid Name** | **Description:** | **Marker** | **Reference** |
| --- | --- | --- | --- |
| pNAT | NAT cassette | ampR | Min *et al*., 2016 |
| pV1093 | Cas9, sgRNA cassette | ampR | Vyas *et al*., 2015 |
| pSN52 | HIS1 cassette | kanR | Noble and Johnson, 2005 |
| pED3 | HIS1-HA-ACT1term cassette | kanR | This study |
| pED20 | WOR1term-NAT cassette | ampR | This study |
| pCR-oplacZ-MDR1-HIS1 | MDR1-HIS1 cassette | ampR | Y. Mao, unpublished data |
| pED10 | pCR-oplacZ-MDR1-HIS1 + SC5314 P_CHT2_-1 | ampR | This study |
| pED11 | pCR-oplacZ-MDR1-HIS1 + SC5314 P_CHT2_-2 | ampR | This study |
| pED12 | pCR-oplacZ-MDR1-HIS1 + SC5314 P_CHT2_-3 | ampR | This study |
| pED13 | pCR-oplacZ-MDR1-HIS1 + P75010 P_CHT2_-1 | ampR | This study |
| pED14 | pCR-oplacZ-MDR1-HIS1 + P75010 P_CHT2_-2 | ampR | This study |
| pED15 | pCR-oplacZ-MDR1-HIS1 + P75010 P_CHT2_-3 | ampR | This study |
| pED17 | pCR-oplacZ-MDR1-HIS1 + SC5314 P_CHT2_-4 | ampR | This study |
| pED18 | pCR-oplacZ-MDR1-HIS1 + SC5314 P_CHT2_-5 | ampR | This study |
| pED19 | pCR-oplacZ-MDR1-HIS1 + SC5314 P_CHT2_-6 | ampR | This study |
| pED21 | pUCIDT + WOR1 PrLD (YF toS) | ampR | This study |
| pED22 | pUCIDT + WOR1 PrLD (DE to A) | ampR | This study |

**REFERENCES**

1 Noble, S. M. & Johnson, A. D. Strains and strategies for large-scale gene deletion studies of the diploid human fungal pathogen Candida albicans. *Eukaryot Cell* **4**, 298-309, doi:10.1128/EC.4.2.298-309.2005 (2005).

2 Russell, C. L. & Brown, A. J. Expression of one-hybrid fusions with Staphylococcus aureus lexA in Candida albicans confirms that Nrg1 is a transcriptional repressor and that Gcn4 is a transcriptional activator. *Fungal Genet Biol* **42**, 676-683, doi:10.1016/j.fgb.2005.04.008 (2005).

3 Min, K., Ichikawa, Y., Woolford, C. A. & Mitchell, A. P. Candida albicans Gene Deletion with a Transient CRISPR-Cas9 System. *mSphere* **1**, doi:10.1128/mSphere.00130-16 (2016).

4 Huang, M. Y., Woolford, C. A., May, G., McManus, C. J. & Mitchell, A. P. Circuit diversification in a biofilm regulatory network. *PLoS Pathog* **15**, e1007787, doi:10.1371/journal.ppat.1007787 (2019).

5 Huang, M. Y., Woolford, C. A. & Mitchell, A. P. Rapid Gene Concatenation for Genetic Rescue of Multigene Mutants in Candida albicans. *mSphere* **3**, doi:10.1128/mSphere.00169-18 (2018).

6 Do, E., Cho, Y. J., Kim, D., Kronstad, J. W. & Jung, W. H. A Transcriptional Regulatory Map of Iron Homeostasis Reveals a New Control Circuit for Capsule Formation in Cryptococcus neoformans. *Genetics* **215**, 1171-1189, doi:10.1534/genetics.120.303270 (2020).

7 Lanni, F. *et al.* Clarifying and Imaging Candida albicans Biofilms. *J Vis Exp*, doi:10.3791/60718 (2020).

8 Fuxman Bass, J. I., Reece-Hoyes, J. S. & Walhout, A. J. Colony Lift Colorimetric Assay for beta-Galactosidase Activity. *Cold Spring Harb Protoc* **2016**, doi:10.1101/pdb.prot088963 (2016).

9 Rupp, S. LacZ assays in yeast. *Methods Enzymol* **350**, 112-131, doi:10.1016/s0076-6879(02)50959-9 (2002).

10 Robinson, J. T. *et al.* Integrative genomics viewer. *Nat Biotechnol* **29**, 24-26, doi:10.1038/nbt.1754 (2011).

11 Shannon, P. *et al.* Cytoscape: a software environment for integrated models of biomolecular interaction networks. *Genome Res* **13**, 2498-2504, doi:10.1101/gr.1239303 (2003).

12 Schindelin, J. *et al.* Fiji: an open-source platform for biological-image analysis. *Nat Methods* **9**, 676-682, doi:10.1038/nmeth.2019 (2012).

13 Lancaster, A. K., Nutter-Upham, A., Lindquist, S. & King, O. D. PLAAC: a web and command-line application to identify proteins with prion-like amino acid composition. *Bioinformatics* **30**, 2501-2502, doi:10.1093/bioinformatics/btu310 (2014).
